# Critical Structural and Functional Roles for the N-Terminal Insertion Sequence in Surfactant Protein B Analogs

**DOI:** 10.1371/journal.pone.0008672

**Published:** 2010-01-13

**Authors:** Frans J. Walther, Alan J. Waring, Jose M. Hernandez-Juviel, Larry M. Gordon, Zhengdong Wang, Chun-Ling Jung, Piotr Ruchala, Andrew P. Clark, Wesley M. Smith, Shantanu Sharma, Robert H. Notter

**Affiliations:** 1 Los Angeles Biomedical Research Institute at Harbor, University of California Los Angeles Medical Center, Torrance, California, United States of America; 2 Department of Pediatrics, Leiden University Medical Center, Leiden, The Netherlands; 3 Department of Medicine, University of California Los Angeles, Los Angeles, California, United States of America; 4 Department of Pediatrics, University of Rochester, Rochester, New York, United States of America; 5 Department of Chemistry and Center for Macromolecular Modeling and Materials Design, California State Polytechnic University, Pomona, California, United States of America; 6 Department of Environmental Medicine, University of Rochester, Rochester, New York, United States of America; University of Giessen Lung Center, Germany

## Abstract

**Background:**

Surfactant protein B (SP-B; 79 residues) belongs to the saposin protein superfamily, and plays functional roles in lung surfactant. The disulfide cross-linked, N- and C-terminal domains of SP-B have been theoretically predicted to fold as charged, amphipathic helices, suggesting their participation in surfactant activities. Earlier structural studies with Mini-B, a disulfide-linked construct based on the N- and C-terminal regions of SP-B (i.e., ∼residues 8–25 and 63–78), confirmed that these neighboring domains are helical; moreover, Mini-B retains critical *in vitro* and *in vivo* surfactant functions of the native protein. Here, we perform similar analyses on a Super Mini-B construct that has native SP-B residues (1–7) attached to the N-terminus of Mini-B, to test whether the N-terminal sequence is also involved in surfactant activity.

**Methodology/Results:**

FTIR spectra of Mini-B and Super Mini-B in either lipids or lipid-mimics indicated that these peptides share similar conformations, with primary α-helix and secondary β-sheet and loop-turns. Gel electrophoresis demonstrated that Super Mini-B was dimeric in SDS detergent-polyacrylamide, while Mini-B was monomeric. Surface plasmon resonance (SPR), predictive aggregation algorithms, and molecular dynamics (MD) and docking simulations further suggested a preliminary model for dimeric Super Mini-B, in which monomers self-associate to form a dimer peptide with a “saposin-like” fold. Similar to native SP-B, both Mini-B and Super Mini-B exhibit *in vitro* activity with spread films showing near-zero minimum surface tension during cycling using captive bubble surfactometry. *In vivo*, Super Mini-B demonstrates oxygenation and dynamic compliance that are greater than Mini-B and compare favorably to full-length SP-B.

**Conclusion:**

Super Mini-B shows enhanced surfactant activity, probably due to the self-assembly of monomer peptide into dimer Super Mini-B that mimics the functions and putative structure of native SP-B.

## Introduction

Lung surfactant is a complex mixture of lipids (mostly phospholipids) and proteins that is required for normal breathing, due to its ability to reduce alveolar surface tension to very low values. Surfactant is synthesized and secreted into the alveolar fluid by type II cells, and consists of approximately 80% phospholipids, 10% neutral lipids and 10% proteins [1], [2]. Despite dipalmitoyl phosphatidylcholine and phosphatidylglycerol constituting its main phospholipid components, the biophysical activity of surfactant in the lung largely depends on the presence of the hydrophobic surfactant protein B (SP-B), and to a lesser degree on the extremely hydrophobic surfactant protein C (SP-C) [3]–[5]. Hereditary SP-B deficiency is lethal in humans [6], while mutations in the SP-C gene may cause interstitial lung disease and increase susceptibility to infection [7]. Surfactant therapy using bovine or porcine lung extracts surfactant extracts, which contain only polar lipids and native SP-B and SP-C, has greatly improved the therapeutic outcomes of neonates with respiratory distress (NRDS). Exogenous surfactant replacement therapies are currently being extended to pediatric and adult patients with direct pulmonary forms of clinical acute lung injury (ALI) and the acute respiratory distress syndrome (ARDS) [8]–[10]. An important goal of surfactant researchers is to replace animal-derived therapies with fully synthetic preparations based on SP-B and SP-C, produced by recombinant technology or peptide synthesis, and reconstituted with selected synthetic lipids (SL) [11]–[16].

**Figure 1:**
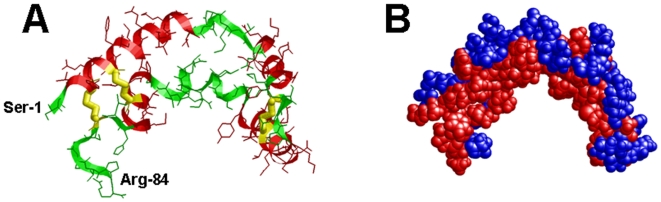
Molecular graphics representations of the three-dimensional (3D) structure of saposin C in the “open” conformation. The 3D-structure of saposin C in SDS detergent in the open conformation was determined earlier from 2D-NMR spectroscopy [20] (PDB accession code: 1SN6; www.rcsb.org). The five helical domains were assigned from DSSP analysis (residues in parentheses) and rendered with Rasmol version 2.7.4.2: alpha-helix-1 (3-19); alpha-helix-2 (26–38); alpha-helix-3 (42–53); alpha-helix-4 (56–61); and alpha-helix-5 (69–75). Plate A: The protein backbone structure is shown with color-coded ribbons denoting alpha-helix (red) or non-alpha-helix (green). Appropriately colored sidechains are shown as stick figures attached to either the alpha-helix (red) or non-alpha-helix (green) ribbon backbones. The N- and C-terminal residues for saposin C (Ser-1 and Arg-84) are indicated at lower left. The three disulfide linkages (i.e., Cys residues 5–78, 8–72 and 36–47) are highlighted in yellow. Plate B: The space-filling model of saposin C is shown in the same orientation as in Plate A. Amino acids (1-letter notation) are indicated as either polar/charged (i.e., E, D, K, R, H, Q, P, N and H) in blue or nonpolar/hydrophobic (i.e., A, T, S, V, G, M, C, I, L, Y, F and W) in red, using the ranking for whole-residue hydrophobicity obtained for free energy calculations at the water-lipid interface [117] (http://blanco.biomol.uci.edu/hydrophobicity_scales.html).

SP-B is a small (79 amino acids; monomer MW of 8.7 kDa), lipid-associating protein that is found in the mammalian lung as a covalently linked homodimer, through a disulfide bridge at positions Cys-51, Cys-51′. Each SP-B monomer contains three intramolecular disulfide bridges (i.e., Cys-8 to Cys-77, Cys-11 to Cys-71 and Cys-35 to Cys-46) [17]. SP-B belongs to the saposin protein superfamily, and earlier X-ray crystallographic or two dimensional nuclear magnetic resonance (2D-NMR) spectroscopic studies on saposins other than SP-B showed that the characteristic saposin fold consists of 4–5 α-helical domains [i.e., ∼residues 10–20 (N-terminal helix), 25–38, 41–52, 59–63 and 68–75 (C-terminal helix)] joined together by 2–3 intramolecular disulfide links [18]. The helical bundle for saposins is folded into two leaves, with one leaf having α-helices 1 and 4–5 and the second leaf composed of α-helices 2 and 3, with flexible hinges between helices 1 and 2 and also between helices 3 and 4–5. For the saposins NK-lysin, granulysin or saposin C in aqueous environments, the protein folds in a *closed* tertiary conformation; here, the two leaves are in close contact such that the amphipathic α-helices with hydrophilic (charged, neutral) residues face the solvent, while the hydrophobic side chains form a core stabilized by intramolecular disulfide bonds [18]. On the other hand, aqueous dimeric saposin B [19] or saposin C bound to submicellar SDS detergent [20] (Fig. 1A, B) show *opened* conformations, in which the leaves of the V are now far apart having expanded at the flexible joints. The respective open conformations allow saposin-B to form noncovalent dimers interacting through their exposed hydrophobic cores [19], while saposin C unmasks its hydrophobic core to bind the fatty acyl chains of SDS detergent [20] (Fig. 1B). These observations support an early proposal [21] that increases in the hydrophobicity of the saposin's environment (e.g., binding to membranes or lipids) may generally produce a greater splay between the protein leaves, thereby exposing more hydrophobic residues.

Although experimental analyses have not yet determined the 3D-structure of full-length SP-B [22], the native protein will probably share the above saposin fold for several reasons. First, the primary sequence of SP-B is highly homologous with those of other known saposins [18], [21], [23]. Second, the intrachain disulfide-linkage pattern observed with SP-B [17] and saposins has been conserved for ∼300 million years [24]. Lastly, circular dichroism (CD) and Fourier transform infrared (FTIR) spectroscopy of native SP-B in membrane mimics indicated high α-helix levels similar to that of other saposins [23], [25]–[27]. Molecular models of homodimeric SP-B, based on templating the primary sequence of SP-B onto the known 3D-structures of NK-lysin, suggested that SP-B may assume the closed and/or opened saposin conformations when interacting with lipid monolayers or bilayers [4], [5], [28], [29]. In the open conformation, the exposed amphipathic helices of SP-B would bind to lipid by inserting its hydrophobic residues to interact with fatty acyl chains, while charged and neutral residues would associate with the more polar lipid headgroup region [5], [30]. Consistent with these proposed SP-B binding models is an early orientation-dependent FTIR study of native SP-B in lipid membranes indicating that a fraction of the helices lie parallel to the lipid surface, while another fraction is slightly embedded in the bilayers, parallel to the fatty acyl chains [25]. It is of particular interest that the above SP-B models predict disk-like structures containing disulfide-linked charged amphipathic helices (i.e., N- and C-terminal domains) which may promote surfactant activity [4], [5], [28].

Previous structural and functional studies with synthetic peptides representing the N- and C-terminal regions of SP-B further support the hypothesis that these charged amphipathic helices participate in surfactant activities. SP-B and a synthetic peptide based on the N-terminal domain of SP-B (i.e., SP-B(1–25); residues 1–25) each increase the collapse pressure of lipid monolayers containing palmitic acid. The cationic N-terminus of SP-B may here interact with anionic lipids to remove the driving force for lipid squeeze-out from the surface film [31], [32]. In additional studies, SP-B(1–25) and native SP-B each induced a coexistence of buckled and flat monolayers when added to surfactant lipids, promoted a low surface tension and increased respreading of the surfactant monolayer [33]. The above *in vitro* surfactant activities of SP-B(1–25) are also correlated with the improved oxygenation and lung compliance noted for this peptide in surfactant-deficient animal models [34]–[37]. Extensive domain mapping experiments have recently confirmed that the N-terminal helical-domain is required for the fusogenic, lytic and surface activities of SP-B [38]. Because physical studies indicated high α-helical levels for N-terminal SP-B peptides in lipids or membrane-mimics [30], [38]–[41], the N-terminal domain in native SP-B may participate in surfactant actions as a charged amphipathic α-helix. Interestingly, several non-natural analogs of SP-B(1–25), which are α-helical in lipid environments, also exhibit *in vitro* surfactant activities [42]. A positively-charged helical C-terminus of SP-B may similarly be involved in lung function, as synthetic peptides representing the C-terminal domain adopt α-helical conformations [43], [44] and promote *in vitro* [43], [45]–[47] and *in vivo* [46], [47] surfactant activities mimicking those of the native protein.

**Figure 2:**
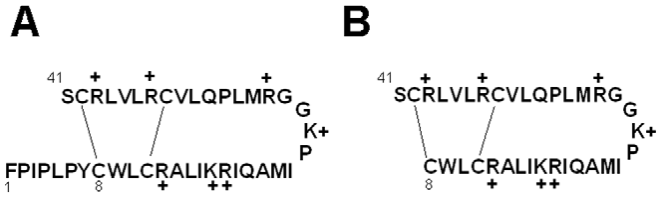
Primary sequences and disulfide bonding patterns for Super Mini-B (S-MB) and Mini-B (MB). Panel A: S-MB (41 amino-acid residues; 1-letter amino-acid notation), with Phe-1, Cys-8 and Ser-41 indicated. Panel B: MB (34 residues), with its numbering based on the parent S-MB, and Cys-8 and Ser-41 indicated. In Panels A and B, the disulfide-linkages are shown between Cys-8 and Cys-41 and between Cys-11 and Cys-34, with the positively-charged arginines (R+) and lysines (K+) denoted. The N-terminal insertion sequence SP-B(1–8) corresponds to NH2-FPIPLPYC-CONH2 (see text).

Because the N- and C-terminal domains are the principal interaction sites for SP-B with surfactant lipids, earlier experiments were performed with an artificial 34-residue construct (i.e., Mini-B) containing these motifs. Mini-B (i.e., MB) incorporates residues 8-25 and 63-78 of native SP-B as a single linear peptide, and was designed to join the critical N- and C-terminal amphipathic helixes with a β-sheet-loop domain [48]. MB folds into a helix-hairpin structure when oxidized, and is stabilized by disulfide connectivity between Cys-8 and Cys-40 and Cys-11 and Cys-34 (residue numbers refer to the MB sequence in Fig. 2B). Conventional ^12^C-FTIR spectroscopy indicated that oxidized MB has elevated α-helical levels in membrane mimics [48], and it is likely that the N- and C-terminal regions will be helical in MB similar to that observed for peptide fragments based on these domains (see above). Indeed, residue-specific analyses using either isotope-enhanced ^13^C-FTIR [48] or 2D-NMR [49] spectroscopy confirmed that MB shares the same three-dimensional saposin fold (see PDB accession codes: 1SSZ and 2DWF; www.rcsb.org) as the predicted full-length SP-B protein in the N- and C-terminal regions [4], [28]. MB in model surfactant lipid mixtures showed marked *in vitro* activity, with spread films exhibiting near-zero minimum surface tension during cycling using captive bubble surfactometry [48]. Using *in vivo* experiments, surfactant-deficient, ventilated rats also demonstrated a rapid recovery of oxygenation (PaO_2_) and dynamic compliance after rescue surfactant treatment with MB and surfactant lipids that was comparable to that of porcine SP-B and lipids [5], [48]. High surfactant activity was additionally determined recently for MB with DEPN-8 (i.e., a novel diether phosphonolipid) employing various *in vitro* techniques [16]. MB had greatly increased adsorption compared to DEPN-8 alone, while MB and DEPN-8 rapidly reached minimum surface tensions in either pulsating bubble or captive bubble surfactometry. In the context of developing fully-synthetic lipid/peptide preparations for treating surfactant deficiencies, it is noteworthy that MB and DEPN-8 mixtures were fully resistant to degradation by phospholipase A2 [16]. In the present paper, we perform similar structural and functional analyses on Super Mini-B or S-MB, an artificial construct that has native SP-B residues (residues 1-7; Phe-Pro-Ile-Pro-Leu-Pro-Tyr) attached to the N-terminus of Mini-B (Fig. 2A), to test whether the N-terminal insertion sequence is also involved in surfactant activity. This putative lipid insertion region of SP-B includes a X-Pro-X-Pro-X-Pro motif and residues (7–9; Tyr-Cys-Trp), and may influence the ability of SP-B to associate with itself [40], [50] or lipids [30], [39], [51], [52]. Consistent with an important function for the N-terminal insertion domain is the finding that the surface activity was reduced for N-terminal peptides with replacements at either tryptophan 9 or prolines 2, 4 and 6 [38], [52]. Moreover, a non-natural analog of SP-B(1–25) with a hydrophobic, helical region substituting for the N-terminal insertion region exhibited more surface activity than the native peptide [53]. The roles that the N-terminal insertion sequence may play in SP-B structure and function were here investigated by comparing the properties of MB and S-MB using a suite of *in vitro* and *in vivo* assays.

## Methods

### Materials

The HBS-EP buffer was from Biacore (Uppsala, Sweden). All organic solvents for sample synthesis were HPLC grade or better.

### Synthesis of Mini-B (MB), Super Mini-B (S-MB) and SP-B(1-8) Peptides

MB (34 amino acid sequence: NH2-CWLCRALIKRIQAMIPKGGRMLPQLVCRLVLRCSCOOH; see Fig. 2B), S-MB (41 amino acid sequence: NH2-FPIPLPYCWLCRALIKRIQAMIPKGGRMLPQLVCRLVLRCS-COOH; see Fig. 2A) and SP-B(1–8) [8 amino acid sequence: NH2-FPIPLPYC-CONH2] were prepared with either a ABI 431A solid phase peptide synthesizer (Applied Biosystems, Foster City, CA) configured for FastMoc™ chemistry [54], a Symphony Multiple Peptide Synthesizer (Protein Technologies, Tucson, AZ) using standard Fmoc synthesis, or a Liberty Microwave Peptide Synthesizer (CEM Corp., Matthews, NC) configured for standard Fmoc synthesis. A low substitution (0.3 mmole/gm) pre-derivatized Fmoc-serine (tBu) Wang resin (NovaBiochem, San Diego, CA) or H-Ser(OtBu)-HMPB Nova PEG resin (NovaBiochem, San Diego, CA) were used to minimize the formation of truncated sequences with the MB and S-MB peptide, while a Rink Amide MBHA resin (NovaBiochem, San Diego, CA) was employed for synthesis of the SP-B(1–8) peptide. All residues were double-coupled to insure optimal yield [48]. After synthesis of the respective linear sequences, peptides were cleaved from the resin and deprotected using a mixture of 0.75 gm phenol, 0.25 ml ethanedithiol, 0.5 ml of thioanisole, 0.5 ml of deionized water and 10 ml trifluoroacetic acid per gram of resin initially chilled to 5°C, and then allowed to come to 25°C with continuous stirring over a period of 2 h to insure complete peptide deprotection [48]. Crude peptides were removed from the resin by vacuum-assisted filtration, and by washing on a medium porosity sintered glass filter with trifluoroacetic acid and dichloromethane to maximize yield. Filtered crude peptides were precipitated in ice cold tertiary butyl ether, and separated by centrifugation at 2000×g for 10 min (2–3 cycles of ether-precipitation and centrifugation were used to minimize cleavage-deprotection byproducts). Reduced crude peptides from ether-precipitation were verified for molecular mass by MALDI-TOF spectroscopy, dissolved in trifluoroethanol (TFE):10 mM HCl (1∶1, v∶v), freeze dried, and purified by preparative HPLC [48]. Final folding of HPLC-purified peptides was facilitated by air-oxidation for at least 48 h at 25°C in TFE and 10 mM ammonium bicarbonate buffer (4∶6, v∶v) at pH 8.0 [55]. Final oxidized MB and S-MB were re-purified by reverse phase HPLC, verified in molecular mass via MALDI-TOF, and disulfide connectivity was confirmed by mass spectroscopy of enzyme-digested fragments (trypsin and chymotrypsin digestion).

### Sodium Dodecyl Sulfate-Polyacrylamide Gel Electrophoresis (SDS-PAGE)

The S-MB and MB peptides were characterized in a detergent environment using sodium dodecyl sulfate-polyacrylamide gel (SDS-PAGE) electrophoresis [56]–[58]. Purified MB or S-MB samples were eluted from reverse-phase HPLC as indicated above. Without either heating or reducing these peptide samples, PAGE with SDS was carried out by dissolving dried peptide (4 µg) into 8 µl of buffer (50 mM MOPS, 50 mM Tris, pH 7.7, 0.1% SDS, 1 mM EDTA; Novex™ 2X buffer from Invitrogen, Carlsbad, CA). PAGE is performed by then applying the dissolved peptide aliquots to precast 16% acrylamide gels (Novex™ Gels, Invitrogen, Carlsbad, CA), which are used because of their reproducibility and minimal sample requirements. Homogenous 16% polyacrylamide gels, which are 43×50×0.4 mm and precast on a 0.175-mm-thick polyester support, are used not only to optimize the resolution in the low molecular weight range, but also to run the two peptides in the same gel under identical conditions. The respective S-MB and MB bands were stained using Coomassie blue and silver to enhance the sample contrast as described previously [56], and bands corresponding to monomeric and oligomeric peptide were identified using tracking molecular weight markers (MW range 2.5–16.9 kDa, Invitrogen, Carlsbad, CA).

### Surface Plasmon Resonance (SPR) Measurements

The respective binding affinities of S-MB and MB, both to themselves (self-association) and to each other (cross-association), were measured with surface plasmon resonance (SPR) spectroscopy using a Biacore 3000 system (Biacore, Uppsala, Sweden) [16], [59]. S-MB and MB, each with a cysteine added to its N-terminus, were chemically linked to a thiol Biacore chip (BR-1000-14, research grade, containing a carboxymethylated dextran matrix covalently attached to a gold film) [60]. Solutions of peptide in HBS-EP buffer (i.e., 10 mM Hepes, pH 7.4, 150 mM NaCl, 3 mM EDTA, 0.005% surfactant P20) were then flowed over the chip-linked peptide at a flow rate of 50 µl/min to determine binding affinity at 37°C. Assessment of peptide binding was determined with sensorgrams, in which the arbitrary response units (RU) were plotted as a function of time. Binding associated with control medium containing no peptide was subtracted from final affinity curves, and mean “on” and “off” rate constants (kon and koff) and the dissociation equilibrium constant (KD = koff/kon) were calculated using BIAevaluation Software Version 4.1 based on curve fitting form measurements at 1 µg peptide/ml buffer concentration. The sensor surface was regenerated using 10 mM HCl between sample injections.

### Prediction of Aggregation-Forming Domains in Peptides

MB and S-MB were each analyzed with PASTA [61], [62] and AGGRESCAN [63], [64] to determine those peptide regions most likely to form β-sheet, particularly when exposed to polar environments such as in the aqueous buffer or at the lipid-water interface [59], [65]. The PASTA algorithm systematically calculates the relative energies of the various pairing arrangements by calculating a pair-wise energy function for residues facing one another within a β-sheet. With a database of known 3D-native structures, PASTA computes two different propensity sets depending on the directionality (i.e., parallel or antiparallel β-sheets) of the neighboring strands. PASTA assigns relative energies to specific β-pairings of two sequence stretches of the same length, and assumes that the lower relative energies enhance aggregation by further stabilizing the cross-β core. The AGGRESCAN algorithm is based on the prior finding that fusion of the amyloid Aβ (1–42) to the green fluorescence protein (GFP) inhibited the folding and fluorescence of GFP by forming Aβ(1–42) aggregates, while Aβ(1–42) mutants that block this aggregation instead promoted fluorescence [66], [67]. Systematic *in vivo* screens of Aβ(1–42) variants fused to GFP permitted the assignment of intrinsic aggregation propensities for natural amino acids, and indicated enhanced aggregation for amino side-chains with increasing hydrophobicity [66], [67]. In conjunction with the experimentally-determined predispositions of amino acids to aggregate and earlier reports that very short sequences may either facilitate or inhibit amyloid fibril formation [68], AGGRESCAN predicts local “hot spots” of aggregation in proteins and peptides other than Aβ(1–42) [63], [64]. Theoretical PASTA and AGGRESCAN predictions of MB and S-MB aggregation were performed here by submitting the primary sequences of these peptides (Fig. 2) to the respective PASTA Version 1.0 (http://protein.cribi.unipd.it/pasta) [62] and AGGRESCAN (http://bioinf.uab.es/aggrescan/) [64] websites.

### Attenuated Total Reflectance-Fourier Transform Infrared (ATR-FTIR) Spectroscopy

Infrared spectra were recorded at 25°C using a Bruker Vector 22 FTIR spectrometer (Pike Technologies) equipped with a DTGS detector, averaged over 256 scans at a gain of 4 and a resolution of 2 cm^−1^ [16], [48]. For spectral measurements of MB and S-MB in hexafluoroisopropanol (HFIP) or methanol (MeOH) solutions, self-films were first prepared by air-drying peptide originally in 100% HFIP onto a 50×20×2 mm, 45° attenuated total reflectance (ATR) crystal for the Bruker spectrometer. The dried peptide self-films were then overlaid with solutions containing 100% HFIP, 40% HFIP/60% deuterated-10 mM sodium phosphate buffer (pH 7.4) or 100% methanol (MeOH) before spectral acquisition; control solvent samples were similarly prepared, but without peptide. Spectra of peptides in solvent were obtained by subtraction of the solvent spectrum from that of the peptide-solvent. For FTIR spectra of MB or S-MB in sodium dodecyl sulfate (SDS), dipalmitoyl phosphatidylcholine (DPPC) or 1-palmitoyl-2-oleoyl phosphatidylglycerol (POPG) environments, lipid-peptide mixtures were spread from chloroform∶trifluoroethanol (1∶1, v∶v) onto the ATR crystal, and then air-dried under nitrogen in the sample chamber to form a multilayer film (SDS∶peptide or phospholipid∶peptide of 40∶1 or 10∶1, mole∶mole, respectively). The peptide∶lipid films were then hydrated with deuterium vapor in nitrogen for 1 h prior to acquiring spectra [40]. The spectra for peptide in SDS, DPPC or POPG were obtained by subtracting the lipid spectrum with D_2_O from that of peptide in lipid with D_2_O hydration. The proportions of α-helix, β-turn, β-sheet and disordered conformations of the resulting IR spectra were determined by self-deconvolution for band narrowing and area calculations of component peaks using curve-fitting programs supplied by Galactic Software (GRAMS/AI 8, version 8.0; Thermo Electron Corp.). The frequency ranges for the different structures were: α-helix (1662–1645 cm^−1^), β-sheet (1637–1613 cm^−1^ and 1710–1682 cm^−1^), β-turns (1682–1662 cm^−1^), and disordered or random (1650–1637 cm^−1^) [69].

### Molecular Modeling of Monomeric MB and S-MB

For molecular modeling and molecular dynamics (MD) simulations of the MB sequence (Fig. 2B), the initial three-dimensional conformation was previously determined from ^13^C-enhanced Fourier transform infrared (^13^C-FTIR) spectroscopy of the disulfide-linked peptide (PDB Accession code: 1SSZ) [48]. The starting MB structure was chosen as the model with lowest energy, least violations of spatial restraints, and the highest number of residues in core regions of the Ramachandran plot. For the corresponding modeling of S-MB, the peptide backbones of the lowest energy conformers of the overlapping SP-B(1–25) (PDB: 1DFW) [40] and MB (PDB: 1SSZ) structures were used as templates for the S-MB sequence (Fig. 2A). The sequences were aligned and homology modeled with Modeller version 9v4 (http://www.salilab.org/modeller/). Both the MB and S-MB structures were further refined using the GROMACS suite of MD programs [70]. The homology structures for MB and S-MB were each placed in a periodic 65 cubic Å box of HFIP∶spc water (4∶6, v∶v) to emulate the solution environment of the FTIR measurements (equilibrated HFIP solvent box and topology files courtesy of D. Roccatano) [71]. The respective ensembles containing either monomeric MB or S-MB peptides were each minimized by the steepest descent method as implemented in the GROMACS version 3.3.3 environment [70] (http://www.gromacs.org). Chloride counterions were added to the solvent box with the peptide to neutralize its charge with constraints on the peptide; the ensembles were then subjected to 100 psec of MD at 300K using the ffG53a6 force field option that allows the solvent to equilibrate while restraining the peptides. These “0 nsec” systems for either MB or S-MB were then subjected to 100 nsec MD simulations at 300K without any experimental constraints, utilizing Berendsen temperature and pressure coupling and the Particle Mesh Ewald method for evaluating long-range electrostatic interactions. The time-dependent evolution of the root mean square deviations (RMSD) for the peptide α-Cs, radius of gyration and secondary structure (i.e., analyzed using the DSSP criteria [72] for the peptide in the HFIP-water environment indicated when equilibrium was reached. Molecular model structures were rendered using Rasmol version 2.7.4.2 (http://www.RasMol.org) and PyMOL v0.99 (http://www.pymol.org).

### Methods for Docking Monomer S-MB to Form Homodimer S-MB

The molecular structure for the S-MB homodimer was derived from the coordinate set generated by the molecular dynamics run of the SMB monomer in HFIP-water. The conformer of S-MB at 49.8 nsec was selected to model the homodimer because its conformation was closest to the β-sheet structure for residues Tyr-7 to Arg-12 as predicted from the PASTA and AGGRESCAN programs (see Results). Two S-MB monomers were initially docked to form a homodimer using ZDOCK [73], similar to that previously described for the docking of the N-terminal domain of SP-B [74]. The lowest energy conformer of this initial SMB homodimeric structure was then further refined by using RosettaDock (www.rosettacommons.org), as implemented in CAPRI [75]. The docking method was performed using a two-step process of rigid-body Monte Carlo searching and parallel optimization of the backbone displacement and side-chain conformations. Monte Carlo minimization was then employed to identify a final lowest energy S-MB dimeric structure [76].

### MD Simulation of the Preliminary S-MB Docked Homodimer in a SDS-Water Environment

Molecular Dynamics (MD) simulations of the S-MB dimer was accomplished by inserting the RosettaDocked peptide homodimer into a pre-equilibrated SDS micelle, which was downloaded from the National Resource for Biomedical Supercomputing (http://www.psc.edu/general/software/packages/charmm/tutorial/mackerell/membrane.html). The ratio of SDS to dimeric peptide was adjusted to 28/1 (i.e., SDS/dimer S-MB) by removing excess detergent. This peptide-SDS ensemble was then minimized in an aqueous 56 Å^3^ solvent box with sodium counter ions for electronic neutrality with Hyperchem 7.5, using the CHARMM 27 option [77]. The coordinate set of this minimized peptide-detergent construct in the PDB format was then ported to the Gromacs environment, and the structure refined using molecular dynamics with the ffG53a6 force field. For the Gromacs environment, the SDS molecule was parameterized using the formalism of Sammalkorpi *et al.* [78]. In this peptide-detergent simulation, the temperature, pressure, electrostatics and bond length constraint run parameters for the molecular dynamics of the system were the same as those used for the monomer S-MB-solvent system (see above).

### Captive Bubble Surfactometry

The captive bubble surfactometer used here was a fully-computerized version of that described by Schurch and co-workers [79]–[81]. In brief, the sample chamber of the apparatus was cut from high-quality cylindrical glass tubing (10 mm inner diameter). A Teflon® piston with a tight O-ring seal was fitted into the glass tubing from the top end, with a plug of buffered 1% agarose gel inserted between the piston and the surfactant solution that was added through a stainless steel port from the other end of the sample chamber. The chamber and piston were vertically mounted in a steel rack, the height of which was regulated by a precision micrometer gear. In a typical experiment, the chamber was filled with a buffered salt solution (140 mM NaCl, 10 mM HEPES, 2.5 mM CaCl_2_, pH 6.9) containing 10% sucrose. One µl of surfactant solution containing 35 µg of lipid was added to this subphase, which was stirred by a small magnetic bar at 37°C. The subphase volume in the sample chamber averaged 0.7 ml (0.5–1 ml), resulting in a final average surfactant lipid concentration of 50 µg/ml (35–75 µg/ml). An air bubble approximately 7 mm in diameter (∼200 µl in volume) was then introduced within the sample chamber and subjected to cyclic volume (surface area) changes by systematically varying the height of the steel rack following a 5 min pause to allow adsorption to the air-water interface. The ionic composition of the buffered agarose plug minimized bubble adhesion to the plug during cycling, so that an uninterrupted bubble interface was maintained. Surface studies utilized a compression ratio of approximately 5∶1 (maximum area/minimum area) and a rate of 20 cycles per min. Bubble images were continuously monitored during compression-decompression using a digital video camera (PULNIX Model TM-200, Pulmix America Inc, Sunnyvale, CA) and a professional video recorder (Panasonic AG-1980P, Secaucus, NJ) coupled to a computer with an Intel Pentium 4 processor. Selected single frames stored in RAM were subsequently subjected to image processing and analysis [82]. Bubble areas and volumes were calculated by an original algorithm relating bubble height and diameter to areas of revolution, and bubble surface tension was determined by the method of Malcolm and Elliot [83].

### Ventilated Lung-Lavaged Rat Model

Animal experiments were performed under established protocols approved by the Animal Care and Use Committee at the Los Angeles Biomedical Research Institute at Harbor-UCLA Medical Center. Anesthesia, surgery, lavage, ventilation, and monitoring methods used have been detailed previously [35]–[37]. Briefly, adult male Sprague-Dawley rats weighing 200–225 g were anesthetized with 35 mg/kg pentobarbital sodium and 80 mg/kg ketamine by intraperitoneal injection, intubated, and ventilated with a rodent ventilator (Harvard Apparatus, South Natick, MA) with 100% oxygen, a tidal volume of 7.5 ml/kg and a rate of 60/min. An arterial line was placed in the abdominal aorta for measurements of arterial blood pressure and blood gases. Rats were paralyzed with 1 mg/kg pancuronium bromide intravenously. Only animals with PaO_2_ values >400 torr while ventilated with 100% oxygen and with normal blood pressure values were included in the experiments. Airway pressures were measured with a pressure transducer (Gould Inc., Cleveland, OH) and tidal volume with a pneumotachometer (Validyne, Northridge, CA) connected to a multi-channel recorder (Gould Inc., Cleveland, OH). The lungs were lavaged 8–12 times with 8 ml of pre-warmed 0.9% NaCl. After the PaO_2_ in 100% oxygen had reached stable values of <100 torr, the rats were treated with 100 mg/kg of experimental surfactant by intratracheal instillation. Arterial blood gases, tidal volume and airway pressures were determined at 15 min intervals throughout each experiment. Dynamic lung compliance was calculated by dividing tidal volume/kg body weight by changes in airway pressure (peak inspiratory pressure minus positive end-expiratory pressure) (mL/kg/cmH_2_O). Ninety minutes after surfactant instillation, rats were killed with 200 mg/kg pentobarbital sodium intravenously. Each treatment group consisted of 8–10 animals.

## Results

### FTIR Spectroscopic Analysis of Mini-B (MB) and Super Mini-B (S-MB) in Lipid Mimic and lipid environments

**Figure 3:**
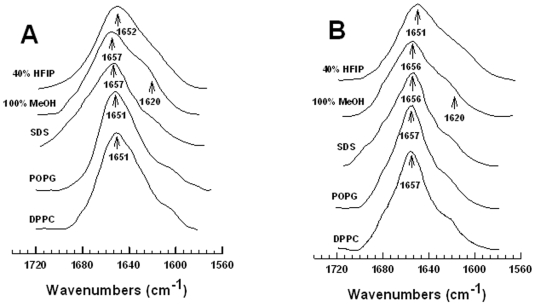
ATR-FTIR spectra of MB and S-MB in the structure-promoting solvents hexafluoroisopropanol (HFIP) and methanol (MeOH), the detergent lipid SDS and the phospholipids POPG and DPPC. Panel A: Stacked FTIR spectra of MB in 40% HFIP (i.e., 40% HFIP/60% deuterated sodium phosphate buffer, pH 7.4), 100% MeOH (i.e., 100% methanol), deuterated SDS, POPG and DPPC. Panel B: FTIR spectra of S-MB in 40% HFIP, 100% MeOH, SDS, POPG and DPPC. In Panels A and B, the IR spectra for MB and S-MB each show dominant α-helical components centered at 1657–1651 cm^−1^ (arrows), with minor bands at ∼1637–1613 cm^−1^ (arrow at 1620 cm^−1^ ) denoting β-sheet. Peptide concentrations were 470 µM for solvent spectra and 10:1 lipid:peptide (mole:mole) for lipid spectra. The abscissa (left to right) is 1740–1560 cm^−1^, while the ordinate represents absorption (in arbitrary units). See text for discussion.

The secondary structures for MB and S-MB in either lipid mimics [i.e., 40% HFIP/60% deuterated-sodium phosphate buffer, pH 7.4 or 100% methanol (MeOH)], lipid-detergent [i.e., sodium dodecyl sulfate (SDS)] or lipids (i.e., POPG or DPPC) were investigated with conventional ^12^C-FTIR spectroscopy. Representative FTIR spectra of the amide I band for MB in these environments (Fig. 3A) were all similar, each indicating a major component centered at ∼1651–1657 cm^−1^ with a small low-field shoulder at ∼1620 cm^−1^. Because prior FTIR studies of proteins and peptides [69], [84] have assigned bands in the range of 1650–1659 cm^−1^ as α-helical, while those bands ∼1613–1637 cm^−1^ reflect β-sheet, MB likely assumes α-helical and β-sheet structures and possibly other conformations in these environments. Self-deconvolutions of the Fig. 3A spectra confirmed that MB is polymorphic, principally adopting α-helix but with significant contributions from β-sheet, loop-turn and disordered components (Table 1). Interestingly, the relative proportions of secondary structure determined for MB (i.e., α-helix > β-sheet ∼ loop-turn ∼ disordered) in both lipids and lipid-mimetics of varying polarity are all comparable (Table 1), suggesting an overall stability for the MB conformation that is well-maintained. In agreement with these results, past ^12^C-FTIR studies of MB in 90% HFIP/10% water [48], or in the lipid-mimetic TFE (pH 7.4) and hydrated multilayers of the synthetic lipid DEPN-8 [16], all showed similar spectra with a major α-helical peak at ∼1655–1658 cm^−1^ and a small β-sheet shoulder at ∼1623 cm^−1^.

**Table 1:** Proportions of secondary structure^a^ for Mini-B and Super Mini-B in structure-promoting solvents (HFIP and methanol), detergent lipid (SDS) and phospholipids (POPG and DPPC), as estimated from self-deconvolution of the ATR-FTIR spectra^b^ of the peptide amide I band.

System	% Conformation			
	α-helix	β-sheet	loop-turn	disordered
*Mini-B*				
40% HFIP	37.5	17.1	25.3	20.1
100% Methanol	34.8	18.8	22.8	23.6
SDS	46.8	14.7	8.4	20.1
POPG	35.6	23.8	21.1	19.5
DPPC	34.5	21.1	23.9	20.5
*Super Mini-B*				
40% HFIP	38.5	21.2	23.9	20.5
100% Methanol	35.2	26.6	29.7	8.5
SDS	37.5	18.5	22.2	21.8
POPG	32.0	24.1	26.7	17.2
DPPC	30.3	26.2	27.3	16.2

a See Fig. 3. Tabulated results are means from four closely-reproduced separate determinations for each condition and spectral type.

b See Methods.

FTIR spectroscopy was also performed to assess secondary structures for S-MB in both lipid-mimic and lipid environments. The representative FTIR spectra obtained for S-MB (Fig. 3B) were similar to the corresponding MB spectra in Fig. 3A with predominate α-helical peaks between 1651–1657 cm^−1^ and a low-field shoulder at 1620 cm^−1^. Deconvolution of these S-MB spectra confirmed elevated levels of α-helix, with smaller amounts of β-sheet, loop-turn and random structures (Table 1). The relative proportions of secondary conformations for S-MB and MB in each of these environments are comparable in Table 1, suggesting that inclusion of the short N-terminal insertion sequence (i.e., S-MB residues 1–7; Fig. 2A) does not grossly perturb the overall secondary conformation of the disulfide-linked core shared by MB and S-MB (Fig. 2). However, it should be noted that somewhat more residues in S-MB were found to participate as β-sheet in these lipid and lipid mimics than those in MB (i.e., 8–11 vs. 5–8 residues, respectively; Table 1). As was observed for MB, the relative percentages of secondary conformations for S-MB were well-conserved in various lipid-mimic and lipid environments (Table 1).

### Molecular Dynamics (MD) Simulations of Monomeric MB and S-MB in a Lipid-Mimic Environment

Molecular dynamics (MD) simulations were next conducted to provide residue-specific information on monomeric Mini-B (MB) and Super Mini-B (S-MB) in the lipid-mimic 40% HFIP/60% water. Although the above ^12^C-FTIR spectroscopic experiments are useful for determining secondary structures averaged over the entire peptide, they cannot indicate the conformations of individual amino-acids. With starting models based on experimental structures, MD runs using GROMACS force-fields should provide worthwhile estimates of the 3D-conformations of both MB and S-MB in lipid-mimics. Here, we performed MD simulations using the ^13^C-FTIR-determined structure of MB in 90% HFIP/10% water (i.e., the 1SSZ structure) [48] as the starting model in 40% HFIP/60% water. The 40% HFIP/60% water environment was chosen for MD simulations for several reasons. First, HFIP (>∼35%) tends to form hydrophobic ‘micellar-like’ clusters in water mixtures [85], [86] that mimic key properties of either detergent micelles or lipid membranes [71], [87], [88]. Second, the FTIR spectra and secondary conformations for MB in 40% HFIP, DPPC or POPG were all similar (Fig. 3A; Table 1), indicating that MD simulations of this peptide in 40% HFIP will be pertinent to its behavior in lipids.

**Figure 4:**
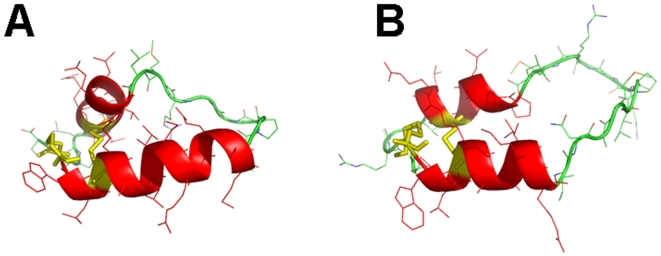
The evolving 3D model of monomeric Mini-B (MB) in 40% HFIP/60% water at the starting (“0 nsec”) and ending (“100 nsec”) times of the molecular dynamics (MD) simulation. Plate A: Snapshot of MB at “0 nsec”. DSSP analysis indicated the following secondary conformation map (residues in parentheses): coil (8, 22, 26–27, 29, 39–41); turn (23–24, 37–38); bend (25, 28); and α-helix (9–21, 30–36) (see text). Plate B: Snapshot of MB at “100 nsec”. DSSP analysis indicated the following conformation map (residues in parentheses): coil (8–9, 18–24, 28–29, 39–41); bend (25–27, 37–38); and α-helix (10–17, 30–36). In Plates A and B, MD simulations were performed in the GROMACS version 3.3.3 environment (see Methods). The protein backbone structure is shown with color-coded ribbons denoting the following domains: N-terminal helix (red), turn-loop (green), and C-terminal helix (red) rendered with PyMOL v0.99. Appropriately colored sidechains are shown as stick figures attached to either the helix (red) or loop (green) ribbon backbones. The orientations of MB in Plates A and B are the same as that for MB in Fig. 2B, with the N-terminal Cys-8 at the far-left bottom. Disulfide linkages between the N-terminal helix in the foreground and C-terminal helix in the background are highlighted in yellow.

**Figure 5 pone-0008672-g005:**
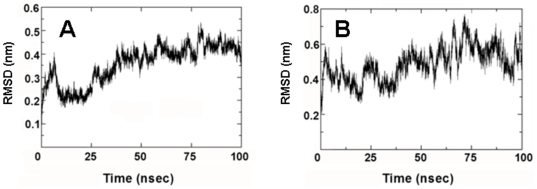
Conformational drift indicated as α-C root mean square deviation (RMSD) from the starting structure for the MD simulation of the monomeric MB and S-MB peptides. Plate A: Time course of the RMSD in nm from the “0 nsec” structure of MB (Fig. 4A). Plate B: Time course of the RMSD from the “0 nsec” structure of S-MB (Fig. 7A). See Methods for experimental details.

**Figure 6 pone-0008672-g006:**
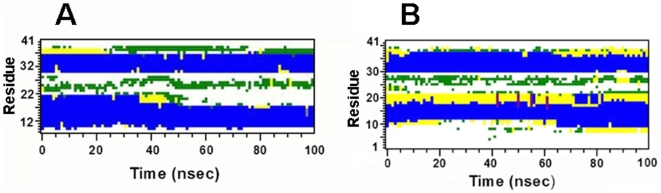
Secondary structure (determined with DSSP [72]) as a function of time for the monomeric MB and S-MB peptides in 40% HFIP/60% water. Plate A: The 34-residue MB peptide. The abscissa range is “0 nsec” to “100 nsec”, while that for the ordinate is 8-41 residues (see Fig. 2B for sequence). Plate B: The 41-residue S-MB peptide. The abscissa range is “0 nsec” to “100 nsec”, while that for the ordinate is 1 to 41 residues (see Fig. 2A for sequence). The secondary structures indicated are: α-helix (blue), 5-helix (purple), 310-helix (grey), turn (yellow), bend (green) and coil (white).

MD simulations were performed on MB by first calculating a “0 nsec” by equilibrating MB in a 40% HFIP/60% water box with chloride counterions (see Methods). This “0 nsec” structure in Figure 4A differs minimally from the 1SSZ structure [48] on which it is based. Fig. 4A indicates that the “0 nsec” model is folded as a helix-hairpin-helix when oxidized, and is stabilized by disulfide linkages between Cys-8 and Cys-40 and Cys-11 and Cys-34 (Fig. 2B). As expected, the axes of the N- and C-terminal helices in the “0 nsec” model are not parallel, but instead are tilted at an angle (Fig. 4A) comparable to that seen in the1SSZ structure [48]. The time course of the adaptation of “0 nsec” MB structure to the lipid-mimetic 40% HFIP was then computed for a 100 nsec-MD simulation, with the final MB model at 100 nsec (i.e., “100 nsec” structure) shown in Fig. 4B. The evolution of the MB structure may be characterized from the kinetics of the root mean square deviation (RMSD) of the α-Cs. The RMSD vs. time plot in Fig. 5A shows that MB in the 40% HFIP environment reaches an equilibrium plateau at ∼40 nsec. The simulations were further studied by examining secondary conformations as a function of time. Figure 6A shows a plot of the MB secondary structure versus time, and indicates that the major conformational elements are largely conserved. For example, the “100 nsec” structure in Fig. 4B confirms the presence of N-terminal α-helix (residues 10–17), loop region with a mix of random coil and bend conformations (18–29) and C-terminal α-helix (30–36). Similar to the “0 nsec” MB structure in Fig. 4A, the “100 nsec” MB model in Fig. 4B folds as a compact N- and C-terminal helical bundle, with considerable interactions between hydrophobic side chains across the interhelix interface. On the other hand, the axes of the N- and C-terminal helices in the “100 nsec” model are now parallel, instead of being tilted at an angle (Fig. 4A) comparable to that seen in either the “0 nsec” or 1SSZ structures [48]. The final “100 nsec” ensemble of MB, HFIP and water molecules also demonstrates that the amphipathic MB peptides sequesters HFIP (not shown), comparably to that previously observed for melittin in HFIP/water mixtures [71], [88].

Partial validation of the “100 nsec” MB structure in 40% HFIP/60% water (Fig. 4B) is provided by our ^12^C-FTIR spectroscopic findings and a previous high-resolution, 2D-NMR structure of MB in detergent micelles. The secondary structures obtained from the deconvolution of ^12^C-FTIR spectra of MB in 40% HFIP (Fig. 3A; Table 1) are broadly compatible with those predicted in the “100 nsec” structure (Fig. 4B). For example, comparably high α-helix was noted in the FTIR spectrum and in the “100 nsec” structure (Table 1). However, the loop-turn and disordered components in the FTIR spectrum (i.e., ∼45% combined in Table 1) are somewhat lower than the roughly-corresponding turn, bend and random coil conformations (i.e., ∼56% combined in Fig. 4B) predicted in the “100 nsec” model. Interestingly, the “100 nsec” MB structure in Fig. 4B did not predict any β-sheet conformation, despite significant β-sheet in FTIR spectra (Fig. 3A and Table 1; see below). The validity of the “100 nsec” MB model may be further tested with an independent 2D-NMR structure determined for MB in SDS-detergent micelles [49] (PDB: 2DWF). Respective overlays of the 2DWF structure with either our “0 nsec” or “100 nsec” MB models indicated much better overlap between the 2DWF and “100 nsec” structures (not shown).

**Figure 7:**
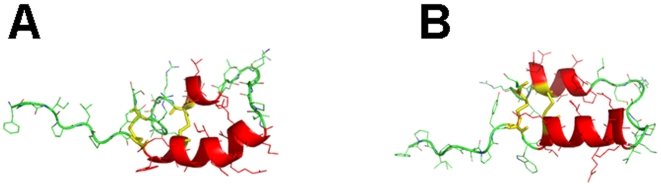
The evolving 3D model of monomeric Super Mini-B (S-MB) in 40% HFIP/60% water at the starting (“0 nsec”) and ending (“100 nsec”) times of the MD simulation. Plate A: Snapshot of S-MB at “0 nsec”. DSSP analysis indicated the following secondary conformation map (residues in parentheses): coil (1–8, 22, 26, 29, 39–41); turn (18–21, 23–25, 34–38); bend (27–28); and α-helix (9–17, 31–33) (see text). Plate B: Snapshot of S-MB at “100 nsec”. DSSP analysis indicated the following secondary conformation map (residues in parentheses): coil (1–6, 23–25, 28–29, 39–41); turn (7–8, 18–21, 36–38); bend (22, 26–27); and α-helix (9–17, 30–35). In Plates A and B, MD simulations were performed in the GROMACS version 3.3.3 environment (see Methods). The protein backbone structure is shown with color-coded ribbons denoting the following domains: N-terminal insertion sequence (green), N-terminal helix (red), turn-loop (green), and C-terminal helix (red) rendered with PyMOL v0.99. Appropriately colored side-chains are shown as stick figures attached to the N-terminal insertion sequence (green), helix (red) or loop (green) ribbon backbones. The orientations of S-MB in Plates A and B are the same as that for S-MB in Fig. 2A, with the N-terminal Phe-1 at the far-left. Disulfide linkages between the N-terminal helix in the foreground and C-terminal helix in the background are highlighted in yellow.

Analogous MD simulations were carried out on S-MB in 40% HFIP. The preequilibration “0 nsec” model (Figs. 6B and 7A) shows that S-MB folds as a helix-hairpin, stabilized by disulfide bonds between Cys-8 and Cys-40 and Cys-11 and Cys-34, with an extended ‘tail’ for the additional N-terminal insertion sequence (Fig. 2A; residues 1-7). DSSP analysis (Figs. 6B and 7A) indicates that the helical bundle region for the “0 nsec” model is substantially different from that of the original 1SSZ structure, with the N- and C-terminal α-helices reduced in length and realigned so that their axes are now parallel. Time-dependent plots of both RMSD (Fig. 5B) and DSSP (Fig. 6B) indicate the S-MB simulation reaches equilibrium at ∼65 nsec, with a final “100 nsec” structure demonstrating a helix-hairpin bundle (Fig. 7B) which closely matches the secondary structure and overall topography of the “100 nsec” MB structure (Fig. 4B). This suggests that the N-terminal insertion domain in S-MB has little influence on the final organization of the helical core, with it projecting away from the N-terminal helix similarly to that of the 1DFW structure of SP-B(1–25) [40]. The N-terminal sequence of S-MB assumes a largely random coil conformation in the “100 nsec” model (Fig. 7B), probably due to its repeated proline motif which prevents back bonding to form intramolecular β-turns and/or β-sheets. The flexible N-terminal tail also contributes to the higher plateau RMSD values observed in Fig. 5B for S-MB than those for MB in Fig. 5A which lacks this sequence (i.e., ∼0.6 and ∼0.4 nm, respectively). Similar to that noted above with the “100 nsec” ensemble for MB, the “100 nsec” S-MB structure is preferentially coated with HFIP selected from the fluorocarbon-water mixture (not shown).

The final “100 nsec” S-MB model (Fig. 7B) obtained from MD simulations was partially corroborated with our ^12^C-FTIR spectral results (Fig. 3B; Table 1). The percent secondary structures obtained from the deconvolution of ^12^C-FTIR spectra of S-MB in 40% HFIP/60% aqueous buffer (Fig. 3B; Table 1) approximate those predicted in the “100 nsec” structure (Fig. 7B), but there remain significant differences. For example, similarly elevated α-helix levels were noted in the FTIR spectrum and in the “100 nsec” structure (Fig. 3B and Table 1). However, the loop-turn and disordered conformations in the FTIR spectrum (i.e., ∼44% combined in Table 1) are reduced from the comparable turn, bend and random coil structures (i.e., ∼63% combined in Fig. 7B) predicted in the “100 nsec” model. It also should be noted that FTIR spectra of S-MB in 40% HFIP indicated ∼21% β-sheet (Fig. 3B; Table 1), while this minor conformation was not identified in the corresponding “100 nsec” structure for S-MB (Figs. 6B and 7B).

### Molecular Weight (MW) and Aggregation Analyses on MB and S-MB Peptides Using SDS-PAGE

**Figure 8:**
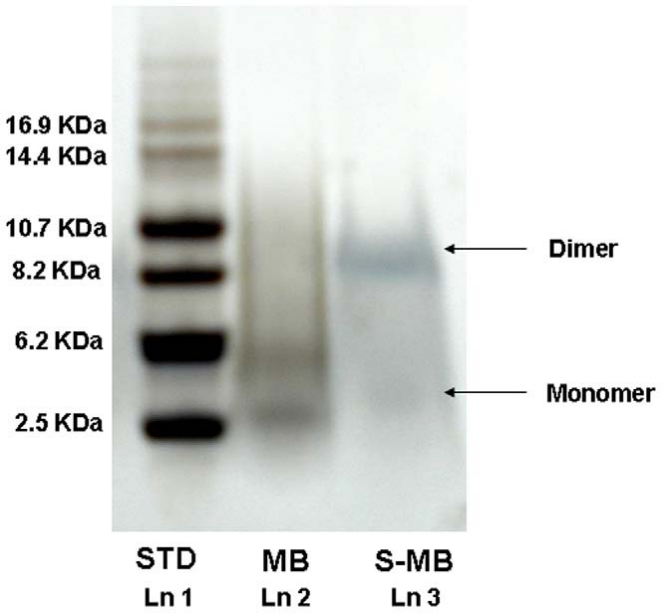
SDS-PAGE analysis of S-MB and MB peptides. Lane 1 (Ln 1): Molecular mass markers (in kDa). Lane 2 (Ln 2): MB, 4 µg. Lane 3 (Ln 3): S-MB, 4 µg. Arrows to the right of Lane 3 indicate the predicted, approximate positions of the respective monomer and dimer bands for MB and/or S-MB. See Methods for additional details.

Both the molecular weight (MW) and self-aggregation properties of MB and S-MB peptides in a lipid-detergent environment were next assessed in sodium dodecyl sulfate-polyacrylamide gel electrophoresis (SDS-PAGE) experiments. MW determinations for proteins and peptides in SDS-PAGE are possible because SDS molecules bind with high affinity to both hydrophobic protein sites [89] and positively-charged amino acid residues, and also because a consistent amount of detergent generally binds to proteins [90]. Maximum levels of SDS bound to proteins (or peptides) occur at ∼1.5–2 g detergent/g protein [91]. Besides MW measurements of monomer proteins, SDS-PAGE has also been useful for investigating the self-aggregation of proteins such as native, full-length SP-B [56], [92]. In our present SDS-PAGE study, MB was initially dissolved in the loading buffer at ∼2 g SDS/g peptide ratio (i.e., ∼27 SDS/MB, detergent to peptide molar ratio), which should be sufficiently high to saturate detergent-binding sites on the peptide. SDS-PAGE was then performed on MB using a protocol that earlier detected native SP-B proteins [56], and Coomassie blue and silver staining showed a diffuse, broad band centered at ∼3.9 kDa (see Figure 8, Lane 2) when compared to the standard molecular mass markers (MW range 2.5–16.9 kDa) (see Figure 8, Lane 1). This MW determined for MB from SDS-PAGE was in good agreement with those assessed from either mass spectrometry (MALDI-TOF) or the known primary sequence, indicating that MB is monomeric on SDS-PAGE. Also in support of the above findings are those from a previous 2D-NMR structural analysis, which demonstrated strong affinity for monomer MB peptide with micellar SDS [49].

SDS-PAGE was similarly performed on S-MB and yielded a broad band at ∼8.9 kDa and a much fainter band at ∼4 kDa (Fig. 8, Lane 3). It is likely that the weak ∼4 kDa band represents low amounts of monomeric S-MB, as it corresponds well with the molecular weights determined from mass spectrometry and the known amino acid sequence. On the other hand, the apparent MW for the principal S-MB band from SDS-PAGE (Fig. 8, Lane 3) is considerably higher than that obtained for monomeric S-MB from mass spectrometry (i.e., ∼8.9 vs. ∼4.7 kDa), and consistent with S-MB forming dimers on electrophoresis. Interestingly, the S-MB peptide, which simulates key structural features of the SP-B leaflet containing the N- and C-terminal α-helical domains and the N-terminal sequence, may also partly mimic the ability of full-length SP-B to form non-covalently associated dimers on SDS-PAGE [92].

### Prediction of Aggregation-Forming Domains in the MB and S-MB Peptides Using PASTA and AGGRESCAN

**Figure 9:**
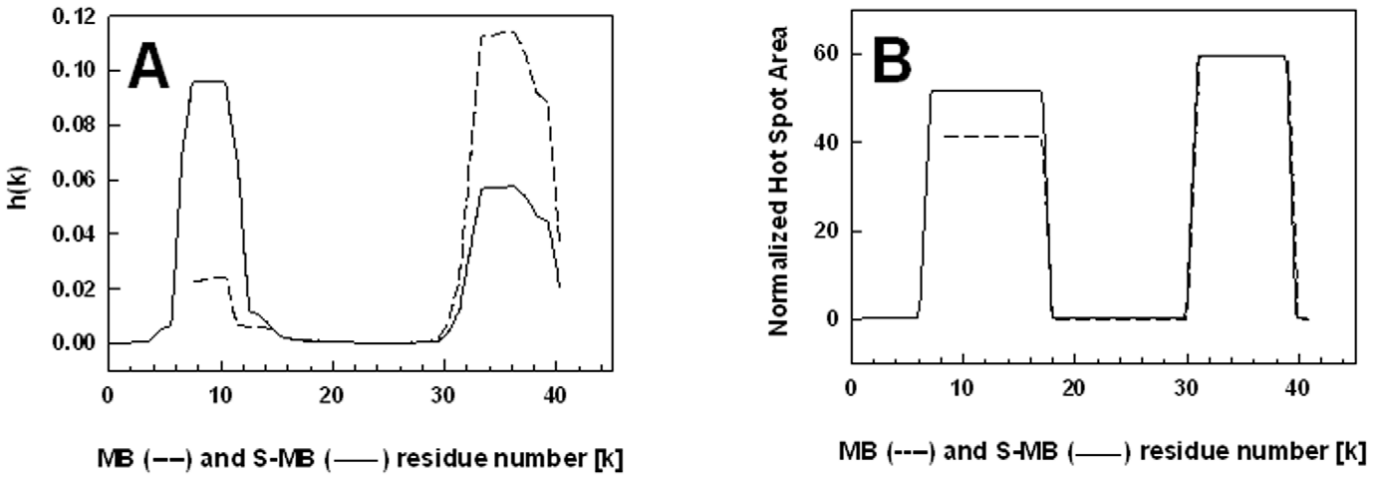
Propensity for β-sheet aggregation determined for MB (----) and S-MB (^____^) from PASTA and AGGRESCAN analyses. Plate A: Plot of the aggregation propensity [i.e., h(k)], calculated from the PASTA algorithm for the relative energies of the various antiparallel and parallel β-sheet pairings [62]. The h(k) values are plotted for each peptide residue (k), and normalized so that the summation of all h(k)s for a peptide equals 1.0. Plate B: Plot of the normalized “hot spot” area as a function of each peptide residue (k), determined from the AGGRESCAN algorithm [64] for the relative propensity of local peptide domains to form aggregates. The sequence and numbering for MB and S-MB are in Fig. 2.

To further explore this differential aggregation of MB and S-MB, PASTA [62] and AGGRESCAN [64] analyses were next performed on these peptides. PASTA predicted that MB will aggregate at both the C- and N-terminal regions. Specifically, PASTA energy calculations showed that the most likely pairing will be an antiparallel β-sheet for the C-terminal residues 34–40 (Fig. 2B), with a relative energy of −5.49. Using as a benchmark a database of 179 peptides derived from the literature [62], an energy threshold of −5.49 indicates that the probability that MB is “amyloid-like” (i.e., highly β-sheet promoting) for this C-terminal segment is ∼90% (true positive rate). Similar PASTA investigations demonstrated that the most likely pairing in the N-terminal region was an antiparallel β-sheet for residues 8–11, with a lower self-aggregation and a higher energy of −5.33 than the corresponding most likely pairing in the C-terminal domain. To account for all possible pairings, a PASTA aggregation profile was next constructed for MB in Fig. 9A [61], and confirmed that segments 34–40 and 8–11 may each form β-sheets; however, this aggregation plot for MB also showed a much lower self-association propensity for the N-terminal domain than that for the C-terminal region (Fig. 9A). An independent analysis of the self-associating domains in MB was similarly conducted with AGGRESCAN, a predictive algorithm which combines the known aggregation tendency of amino acids with earlier findings that short sequences (∼5 residues) either promote or inhibit interpeptide β-sheets (see Methods). The AGGRESCAN aggregation profile of MB is shown in Fig. 9B, in which the “normalized hot spot area” (i.e., directly proportional to the self-association propensity of a residue) is plotted as a function of each peptide residue (k). The AGGRESCAN plot for MB in Fig. 9B confirms our PASTA predictions in Fig. 9A that not only will the N- and C-terminal domains of MB be self-association zones, but also that the C-terminal region will exhibit a higher aggregation tendency than the N-terminal domain. These PASTA and AGGRESCAN computations suggest that the β-sheet conformations identified in the FTIR spectra of MB (Fig. 3A; Table 1) are due to interpeptide β-sheets forming between opposing amino-acids of the N-terminal (∼ residues 8–11) and/or C-terminal (∼residues 34–40) domains. Nevertheless, these interpeptide interactions are apparently too weak to promote oligomers for MB on SDS-PAGE, as only the monomer was observed in Fig. 8.

PASTA and AGGRESCAN calculations were next conducted on S-MB, and indicated that attachment of the N-terminal insertion sequence to the MB peptide dramatically enhanced the self-association properties of the N-terminal region. For example, PASTA analysis of S-MB demonstrated that an antiparallel β-sheet for the N-terminal segment 7–12 (Fig. 2A) is now the most likely pairing for the entire S-MB peptide. The PASTA energy for the antiparallel β-sheet segment for S-MB is −6.07, significantly lower than that of the most likely pairing of the C-terminal domain of S-MB (i.e., segment 34–40) with a relative energy of −5.49. Inclusion of the N-terminal insertion sequence increases the probability that S-MB is “amyloid-like” (i.e., highly β-sheet) to ∼97% from the ∼90% value determined above for MB [62]. The PASTA aggregation plot for S-MB in Fig. 9A confirms that the N-terminal region (∼ residues 7–12) now shows a predominant self-association propensity over that determined for the C-terminal region (∼ residues 34–40). A similar AGGRESCAN aggregation profile for S-MB in Fig. 9B also shows that the self-association properties of the N-terminal region (∼ residues 7–16) are increased over those noted for the truncated MB peptide, although here the self-association of the C-terminal region (∼ residues 32–38) is predicted to be slightly higher than that of the N-terminal domain. Taken together, these PASTA and AGGRESCAN results suggest that the dimer formation observed for S-MB, but not for MB, in Fig. 8 is due to S-MB possessing the Tyr-7 residue in the N-terminal insertion sequence (1–7). Nevertheless, our subsequent MD simulations of dimer S-MB in a SDS-water environment indicated that the enhanced self-association observed with S-MB is not simply due to the inclusion of Tyr-7, but is also the result of the Phe-1 to Pro-6 sequence participating through a distinct mechanism (see below).

One cautionary note in using the above PASTA and AGGRESCAN algorithms is that they do not account for the actual solvent environment of a given protein domain. Thus, these predictions may not be used to directly calculate association constants and binding stoichiometries, which require more precise binding free energies [93]. Instead, relative aggregation ‘propensities’ are assessed for protein sequences with PASTA or AGGRESCAN, each using databanks to benchmark a given domain [62], [64]. These aggregation propensities are most accurate for peptide regions exposed to polar environments [65], such as in aqueous buffer or the membrane lipid-water interface [59], but are less applicable for proteins in hydrophobic milieu. In this context, it is worthwhile analyzing the lung SP-C (see Introduction) with these predictive algorithms. SP-C is a hydrophobic 35-amino acid transmembrane protein that not only exhibits lung surfactant activities, but also is associated with the onset of pulmonary alveolar proteinosis (PAP). Bronchoalveolar fluid from PAP patients is rich in insoluble SP-C aggregates, which show amyloid properties such as Congo red staining and fibril formation on electron microscopy [94]. PASTA and AGGRESCAN further support an amyloid classification for SP-C, as both predict extremely high aggregation with relative PASTA energies and normalized hot spot areas of −29.2 (SP-C residues 8-28 as parallel β-sheet) and 128 (SP-C residues 12-35 as β-sheet) (Gordon *et al.*, unpublished observations). Consequently, PASTA and AGGRESCAN successfully predict only the high β-sheet detected with FTIR spectra of depalmitoylated SP-C in aqueous buffer at neutral pH [95], but not the elevated α-helix found in CD spectra of this peptide in detergent micelles [96]. Because MB and S-MB are each likely exposed to water when bound to lipids at the polar headgroup region [30], [49], [97], we anticipate that our PASTA and AGGRESCAN results in Fig. 9 will also accurately forecast aggregated domains in MB and S-MB. These predictions also permitted us to develop starting dimer S-MB models for MD simulations to more rigorously assess protein-protein interactions (see below).

### Surface Plasmon Resonance (SPR) Measurements of MB and S-MB

**Figure 10:**
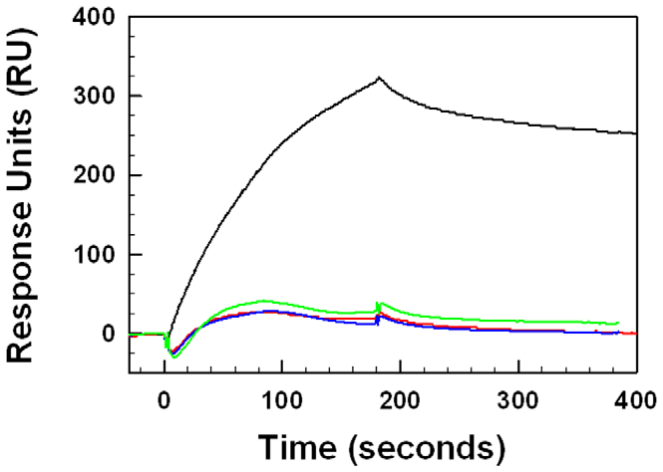
Surface plasmon resonance (SPR) sensorgrams of S-MB or MB binding to immobilized Cys-S-MB or Cys-MB. S-MB and MB, each with a cysteine added to its N-terminus, were attached to the thiol Biacore chip as described in the text. Solutions of S-MB or MB in HBS-EP buffer (i.e., 10 mM Hepes, pH 7.4, 150 mM NaCl, 3 mM EDTA, 0.005% surfactant P20) were then flowed over the respective chip-linked peptides. Typical SPR responses [Y-axis indicates the relative amount of binding in arbitrary response units (RU)] are shown for either 1 µg S-MB/ml buffer to chipped Cys-S-MB (black line) or chipped Cys-MB (green line), or with 1 µg MB/ml buffer to chipped Cys-S-MB (red line) or Cys-MB (blue line). Relative peptide affinities are: S-MB to S-MB ≫ S-MB to MB∼ MB to S-MB ∼ MB to MB.

Given the differential self-aggregation observed for S-MB and MB on SDS-PAGE (Fig. 8), it is important to determine the direct binding affinities of these peptides, both to themselves (self-association) and to each other (cross-association). Here, we use surface plasmon resonance (SPR) spectroscopy to make these peptide-peptide binding assessments. SPR is a surface-sensitive methodology, in which the ligand is chemically-linked to a sensor surface, and the solute is then flowed past the “chipped” molecule. The binding of the solute to the immobilized ligand produces an evanescent response, which is measured in response units (RU) and is proportional to the bound mass. SPR experiments have recently determined the self-association properties of amyloid peptides [98], which bear some similarities to our synthetic surfactant peptides (see above). In the present studies, S-MB and MB were each chemically-linked to chips at their respective N-terminal amine groups, while the solutes S-MB and MB in buffer were flowed past the immobilized peptides. Molecular binding-affinities (associations) between the soluble peptides and chip-linked films of S-MB and MB were measured at 37°C using a Biacore apparatus. Representative sensorgrams of S-MB and MB binding to chipped S-MB and MB peptides are shown in Fig. 10, and indicate that the self-association of S-MB is considerably greater than that observed for MB. Specifically, plots of RU as a function of time indicated a much higher maximal RU value for 1 µg S-MB/ml buffer flowed past chipped S-MB than that for 1 µg MB/ml buffer flowed over chipped MB (i.e., respective maximal RU values of 325 and 28). Also consistent with a higher self-association for S-MB than that for MB is the much lower dissociation constant (KD) observed for S-MB binding to chipped S-MB than the corresponding KD for MB binding to chipped MB (Table 2). Control SPR experiments examining the binding of S-MB to chipped MB, and also MB to chipped S-MB, produced not only low-response sensorgrams which nearly overlap that obtained for MB binding to chipped MB (Fig. 10), but also higher KD values than that observed for S-MB binding to itself (Table 2). These SPR results indicating much higher self-association for S-MB than that noted for MB are supportive of our SDS-PAGE findings indicating dimer formation for S-MB but not for MB (Fig. 8).

**Table 2:** Mean association and dissociation kinetic rate constants (k_on_, k_off_) and equilibrium dissociation constants (KD), calculated from surface plasmon resonance (SPR) measurements for aqueous Mini-B (MB) and Super Mini-B (S-MB) flowing past chip-linked Cys-MB and Cys-S-MB monolayers^a^.

Aqueous peptide	Chip-linked peptide	k_on_ (1/Ms)	k_off_ (1/s)	KD (nM)
MB	MB	9.19×10^4^	1.68×10^−2^	183.0
MB	S-MB	2.00×10^5^	8.06×10^−3^	40.4
S-MB	MB	1.41×10^5^	7.36×10^−3^	52.1
S-MB	S-MB	4.47×10^4^	9.01×10^−4^	20.1

a MB and S-MB in running buffer (10 mM HEPES, 150 mM NaCl, 3 mM EDTA, 0.005% Surfactant P20, pH 7.4) were flowed past monolayers of N-terminal Cys-MB or N-terminal Cys-S-MB, linked via their respective N-terminal thiol groups to CSM sensor chips in a Biacore 3000 system (Methods).

Mean kinetic rate constants (k_on_, k_off_) and equilibrium dissociation constants (KD = k_off_/k_on_) were determined from curve fitting analyses of SPR results at 1 µg peptide/ml buffer.

### Prediction of Dimer S-MB Structures Using ZDOCK and RosettaDock

**Figure 11:**
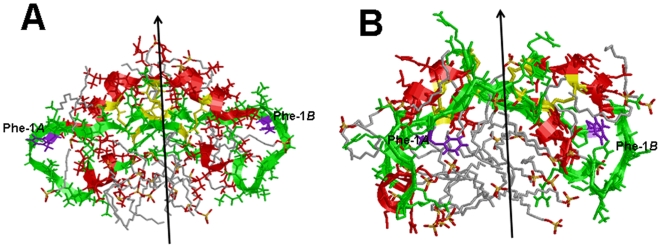
The evolving 3D model of dimer Super Mini-B (dimer S-MB) in SDS/water at the starting (“0 nsec”) and ending (“10 nsec”) times of the MD simulation. Plate A: Snapshot of dimer S-MB at “0 nsec” (see text). DSSP analysis indicated helical regions (residues in parentheses) for S-MB molecules *A* (14–17, 30–37) and *B* (14–18, 31–39). The local 2-fold axis relating the two monomers in the dimer is shown by an arrow. Plate B: Snapshot of S-MB at “10 nsec”. DSSP analysis indicated helical regions for S-MB molecules *A* (8–10, 17–21, 30–37) and *B* (11–16, 31–38). In Plates A and B, MD simulations were performed in the GROMACS version 3.3.3 environment (see Methods). The protein backbone structure is shown with color-coded ribbons denoting the following domains: N-terminal insertion sequence (green), N-terminal helix (red), turn-loop (green), and C-terminal helix (red) rendered with Rasmol 2.7.4.2. Appropriately colored side-chains are shown as stick figures attached to the N-terminal insertion sequence (green), helix (red) or loop (green) ribbon backbones. Disulfide linkages between the N-terminal helix in the foreground and C-terminal helix in the background are highlighted in yellow. The helices are predominately α-helical, with additional minor contributions from 3_10_ - and 5-helices. The side-chains and backbones for the two N-terminal phenylalanines are colored purple. The N-terminal sequences (residues 1–7) adopt extended conformations that are centered just over the N- and C-terminal helices, with each having its N-terminal Phe-1 near the loop region (Gly-25 and Gly-26). The 30 bound SDS detergent molecules are shown as wireframe molecules that are colored according to the cpk convention. The “0 nsec” dimer S-MB structure in Plate A is similar to the initial ZDOCK and Rosetta input structures (see text).

Because S-MB predominately formed dimers in the above SDS-PAGE (see above), potential 3D-interactions between S-MB monomers were next assessed with several docking algorithms. ZDOCK is a preliminary stage docking program, which optimizes shape complementarity, desolvation and electrostatics using Fast Fourier Transform (FFT) based methods [73], [99]. Rather than allow flexibility in the surface side-chains and/or backbones of the two proteins, ZDOCK uses searches that permit the proteins to interact only as rigid-bodies [99]. With S-MB, this *soft docking* approach may be particularly useful, given that FTIR spectroscopy (Fig. 3; Table 1) and MD simulations of the monomer (Figs. 4–7) indicate that the disulfide-linked N- and C-helical domains are stable in a wide-range of lipid-mimic and lipid environments. Because PASTA and AGGRESCAN programs both predict that the S-MB sequence containing residues Tyr-7 to Arg-12 is the most stable β-sheet pairing, initial ZDOCK searches were performed with a 49.8 nsec simulation of the MD run of monomer S-MB in 40% HFIP/60% water. As indicated in Fig. 6B, the S-MB sequence 7–12 adopts an extended conformation that most closely approximates that of extended β-sheet. In light of PASTA further predicting that the lowest relative energy will be an antiparallel β-sheet for residues 7–12, ZDOCK searches were next conducted with the dimer S-MB folded as an antiparallel β-sheet for residues ∼7–12. The lowest energy conformer for the resulting S-MB homodimer maintained a close antiparallel apposition between the two Trp-Leu pairings (i.e., Trp-9*A* to Leu-10*B* and Leu-10*A* to Trp-9*B*; *A* and *B* referring to the two S-MB sequences in the homodimer). An approximate two-fold axis lies through the center of the two Trp-Leu pairings that relates the two “leaves” of the S-MB dimer. Each of these leaves includes the helical bundle containing the N- and C-terminal α-helices, and also the N-terminal insertion sequence (1-7) that adopts an extended conformation near the N- and C-terminal helices (see Fig. 11A). Last, the leaves appear to splay about the local dyad in a relatively open conformation.

The lowest energy conformer from the above ZDOCK search for S-MB homodimers was then further analyzed by using RosettaDock [100], as implemented in CAPRI [75]. Although ZDOCK permits fast global docking searches, its use of course-grained representations for proteins or peptides may not be an accurate model of the binding surfaces [100]. The RosettaDock methodology uses a two-step process of rigid-body Monte Carlo searching and parallel optimization of the backbone displacement and side-chain conformations. Here, a plot of the energies of 1000 S-MB homodimers *versus* the RMSD from the initial ZDOCK conformation was produced by the RosettaDock server. This plot indicated that the top ten scoring (i.e., lowest energy) candidates reside in a ‘docking funnel’ near the starting input conformation (not shown). Thus, RosettaDock converged to final S-MB dimer models similar to the lowest energy conformer from ZDOCK. Indeed, the overall folding pattern of the lowest-energy RosettaDock model for the S-MB dimer closely reproduced that of the lowest-energy ZDOCK dimer structure (see above and Fig. 11A).

### MD Simulations of Dimer S-MB with SDS in Water

It is of considerable interest to determine whether homodimeric S-MB structures similar to those identified in the above docking studies are also present in the SDS-PAGE experiments. MD simulations on dimer S-MB were conducted here by first inserting the lowest energy conformer from the above RosettaDock computations into a SDS-water mileu with a detergent/peptide molar ratio of 28/1. This peptide-SDS ensemble was then minimized in an aqueous solvent box with Hyperchem 7.5, using the CHARMM 27 option (see Methods). The environment for the resulting “0 nsec” structure of dimer S-MB thus closely approximates that of our SDS-PAGE experiments, which similarly use a loading buffer containing submicellar SDS at a detergent/peptide molar ratio of ∼28/1 (Fig. 8).

The “0 nsec” dimer S-MB model in Fig. 11A shows a peptide structure comparable to those described above for the lowest energy conformers in either the ZDOCK or RosettaDock studies of dimer S-MB. Specifically, the “0 nsec” homodimer exhibits a near antiparallel juxtaposition between the two Trp-Leu pairings of the monomers. An approximate two-fold axis lies through the center of these Trp-Leu pairings, which relates the two “leaves” that contain not only the N- and C-terminal helical bundles, and also the extended N-terminal insertion sequences (residues 1–7) (Fig. 11A).

Secondary structure analysis of the “0 nsec” dimer S-MB structure demonstrates that the N- and C-terminal regions largely maintain their helical conformations, but that there is some fraying of the two N-terminal helices at residues Arg-12 to Leu-14. The topographical organization of the S-MB dimer in Fig. 11A indicates that the two leaves are in an extended, or “open”, conformation that is readily accessible from all sides to SDS molecules and water. In the “0 nsec” dimer S-MB structure of Fig. 11A, anionic SDS closely associates not only with exposed nonpolar residues through hydrophobic interactions, but also with positively-charged Arg and Lys residues through electrostatic interactions. The binding of SDS to the “0 nsec” dimer S-MB appears to be concentrated in two surface cavities near the center of the dimer peptide in Fig. 11A, and these two shallow depressions containing SDS are themselves related by the aforementioned two-fold axis.

**Figure 12:**
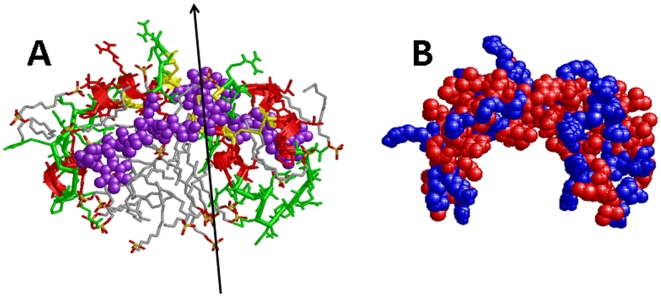
Molecular graphics representations of the 3D structure of dimer S-MB after 10-nsec of MD simulation in a SDS/water environment. The 3D-structure of dimer S-MB the open conformation was determined from the 10-nsec MD simulation of a ZDOCK/RossettaDock structure, with 28 SDS molecules in a water box and rendered using Rasmol version 2.7.4.2 (see Methods). Plate A: The protein backbone structure is shown with color-coded ribbons denoting helix (red) or non-helix (green), with appropriately colored sidechains indicated as stick figures. The four disulfide linkages are highlighted in yellow. The two N-terminal insertion sequences (residues 1–7) are represented by space-filling backbones and sidechains colored in purple. These N-terminal sequences adopt extended conformations that are centered just over the N- and C-terminal helices, with each having its N-terminal Phe-1 near the loop region (Gly-25 and Gly-26). The 28 SDS lipids are shown as wireframe molecules that are colored according to the cpk convention. The approximate two-fold axis relating the two monomers is shown by an arrow. Plate B: The space-filling model of dimer S-MB is shown in the same orientation as in Plate A, but without the SDS detergent. Polar/charged and nonpolar/hydrophobic residues are colored in blue and red, using the Wimley-White ranking system [117] (see Fig. 1B).

The time course of the adaptation of “0 nsec” dimer S-MB in SDS and water was then calculated for a 10 nsec-MD simulation, with the final dimer S-MB model at 10 nsec (i.e., “10 nsec” structure) shown in Fig. 11B. The “10 nsec” model indicates that the dimer S-MB converts to a oblate spheroid from the original, more extended “0 nsec” model, with a concomitant merger of the two shallow cavities into a single deep cavity that is exposed to the external solvent (Fig. 11). The interior of this central cavity in dimer S-MB is now lined with hydrophobic amino-acid sidechains capable of interacting with hydrophobic detergents such as SDS. Fig. 11B shows that the SDS molecules primarily occupy the central cavity, and are oriented so that their anionic headgroups either face the exterior water or form ion pairs with the cationic residues (i.e., Arg, Lys) that line the outer periphery of the cavity. The fatty acyl groups of SDS in this cavity are also directed towards the protein interior. Space-filling models (not shown) confirm that the “10 nsec” dimer S-MB folds as a globular lipoprotein, consisting of a partial ‘micelle’ of ∼25 SDS molecules in the central cavity and the dimer peptide providing the remainder of the structure. Analogous to other thermodynamically-stable, soluble proteins, the surface of the “10 nsec” structure is enriched in charged or polar molecules (e.g., positively-charged amino-acids or anionic sulfates of SDS), while the protein interior is dominated by hydrophobic groups (e.g., nonpolar amino acids or hydrocarbon tails of SDS molecules) (see Fig. 11B). The local two-fold axis identified in the “0 nsec” structure (Fig. 11A) is conserved in the “10 nsec” structure (Figs. 11B and 12A), and the “10 nsec” dimer S-MB model also demonstrates secondary conformations comparable to those seen in the “0 nsec” model, although with additional fraying of several helical elements (Fig. 11).

A RMSD vs. time plot shows that the MD simulation of the entire dimer S-MB ensemble reaches rapid equilibrium and a plateau phase in ∼3 nsec, confirming that the dimer S-MB-SDS complex is stable in water for the 10 nsec run. The reorganization of bound SDS likely provides the thermodynamic driving force for the conversion of the dimer S-MB-SDS complex to the oblate spheroid conformation (i.e., “10 nsec” structure”) in Fig. 11B. For example, the separation between the two antiparallel segments Tyr-7 to Arg-12 in the “0 nsec” structure is dramatically increased in the “10 nsec” structure (see top of Fig. 11). Two SDS molecules insert between these two opposing strands in the “10 nsec” model, thereby increasing by ∼10 Å the α-C distance between the Trp-9*A* and Trp-9*B* residues. This restructuring of bound SDS is also probably responsible for the lengthwise narrowing of the central portion of dimer S-MB, in which the distance between the α-Cs of Phe-1*A* and Phe-1*B* decreases from 42.69 Å (“0 nsec” model) to 38.01 Å (“10 nsec” model). Simultaneous with this narrowing is the creation of the central cavity in the “10 nsec” dimer S-MB model (Fig. 11). In the “0 nsec” or “10 nsec” models of Figs. 11 and 12A, the two leaves in dimer S-MB each consist of the N- and C-terminal helical bundle and the N-terminal sequence (1–7), and are related to one another by a local two-fold axis. In the “10 nsec” model, however, the two leaves form the walls of the internal cavity that hold the SDS detergent. The effective clamping down on the bound SDS molecules by the dimer S-MB leaves in the “10 nsec” structure is probably due to strong hydrophobic interactions between the SDS detergent molecules and the nonpolar sidechains that project from the backbones of the helical bundle and the N-terminal sequence in the interior of the cavity (Fig. 12A). Also contributing to the stability of the dimer S-MB complex are the Arg and Lys residues at the surface periphery of the cavity, which form electrostatic interactions between the negatively-charged headgroups of SDS and the positively-charged Arg (residues 12*A*, 17*A*, 12*B* and 17*B*) and Lys (16*A*, 24*A*, 16*B* and 24*B*). The space-filling model for dimer S-MB in Fig. 12B shows more clearly how the hydrophobic residues are clustered on the surfaces of the interior cavity, while polar and charged residues line the periphery of this hydrophobic region. Given that the present “10 nsec” model for dimer S-MB in Fig. 11B was developed with MD simulation conditions approximating those of our SDS-PAGE experiments, this structure may also be responsible for the dimer band observed in our electrophoresis studies (Fig. 8). Last, it is of interest to note that that our final “10 nsec” model for the docked S-MB dimer adopts a ‘saposin-like’ conformation (Figs. 11B and 12) that bears a striking resemblance to the 3D-structure of saposin C in SDS that was determined using 2D-NMR spectroscopy (Fig. 1) [20] (see Discussion).

### *In Vitro* Dynamic Surface Activity

**Figure 13:**
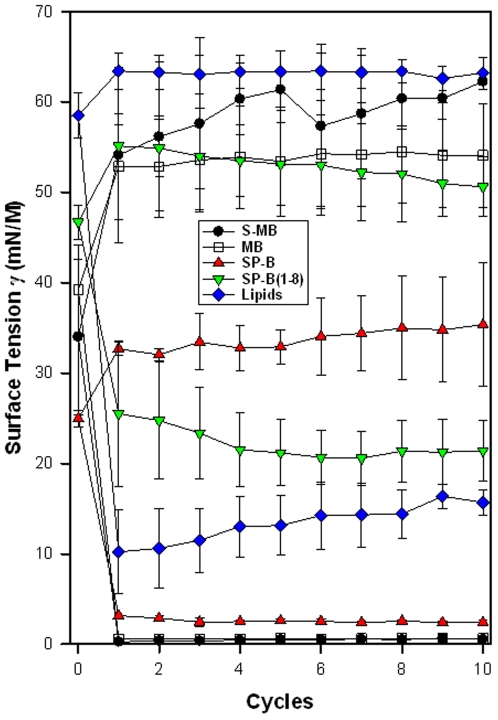
Surface activity of synthetic surfactants and native SP-B on the captive bubble surfactometer. Minimum and maximum surface tension values are plotted for synthetic lipids with 1.5% by weight Mini-B [MB], Super Mini-B [S-MB], SP-B(1-8), or native pig SP-B, and SL alone for 10 successive compression-expansion cycles on a captive bubble surfactometer (20 cycles/min, 37°C). Synthetic lipids = 16:10:6:1:2 (weight ratio) DPPC:POPC:POPG:POPE: cholesterol. Values shown are mean ± SEM for n = 4.

Synthetic surfactant preparations were formulated by mixing synthetic lipids (SL), consisting of 16∶10∶6∶1∶2 (weight ratio) DPPC∶POPC∶POPG∶POPE∶cholesterol, with 1.5 mol% S-MB, MB, SP-B(1–8), or native pig SP-B. S-MB and MB surfactants had very high surface activity in captive bubble experiments and reached identical minimum surface tension values <1 mN/m during each of ten consecutive cycles of dynamic cycling (rate of 20 cycles/min, Figure 13). Pig SP-B surfactant (positive control) reached minimum surface tension values <3 mN/m, whereas SP-B(1–8) surfactant, based on the SP-B insertion sequence which is present in S-MB and absent in MB, and lipids alone (negative control) reached significantly higher minimum surface tension values of 20 and 16 mN/m (p<0.001 versus S-MB, MB and pig SP-B surfactants) after ten cycles on the captive bubble surfactometer.

### *In Vivo* Activity of Synthetic Surfactants in Ventilated, Lung-Lavage Rats with ARDS

**Figure 14:**
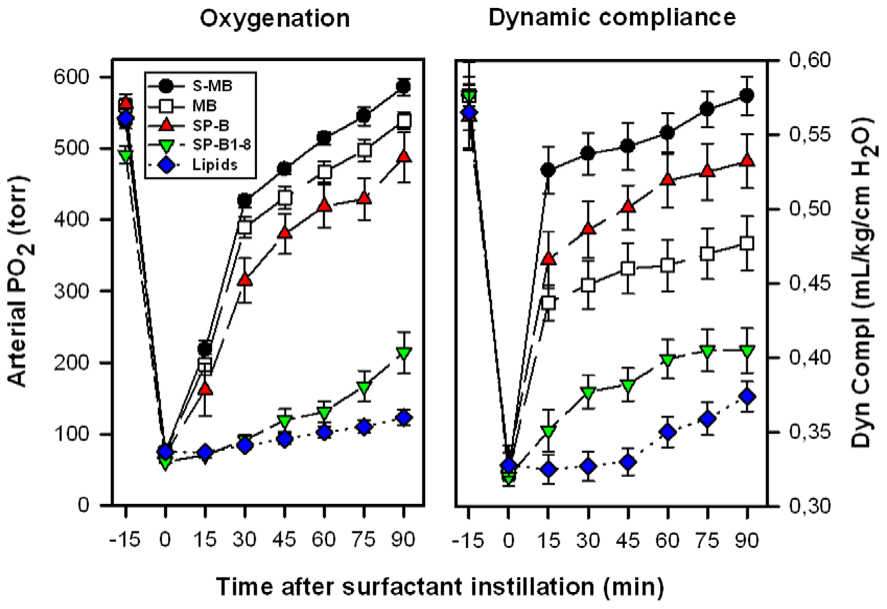
Arterial oxygenation and dynamic compliance in surfactant-treated, ventilated rats with ARDS induced by *in vivo* lavage. Arterial partial pressure of oxygen (PaO_2_ in torr) and dynamic compliance (mL/kg/cm H_2_O) are shown as a function of time for groups of 8–10 rats treated with synthetic lung surfactants (synthetic lipids +1.5 mol% S-MB, MB, or SP-B(1–8)), synthetic lipids +1.5% porcine SP-B (positive control), or synthetic lipids alone as a negative control. Data are shown as mean ± SEM.

The pulmonary activity of S-MB and MB surfactant (described above) was investigated in comparison to native pig SP-B (positive control) and SP-B(1–8) surfactants and lipids alone (negative control) during a 90 min period following intratracheal instillation of these surfactants into ventilated rats with ARDS induced by *in vivo* lavage. Oxygenation and lung compliance (Figure 14) increased quickly after instillation of S-MB, MB, and pig SP-B surfactant. Instillation of the negative control of lipids alone or SP-B(1–8) surfactant had minimal effects on arterial oxygenation or compliance. The relative order of pulmonary activity in terms of both oxygenation and compliance was given as: S-MB > MB > pig SP-B > SP-B(1–8)> lipids alone (negative control) (Figure 14). The differences in oxygenation and compliance between S-MB, MB and pig SP-B surfactants were statistically significant (p<0.05) starting at 30 min after surfactant instillation and consistently surpassed the performance of SP-B1-8 and lipids only surfactants (p<0.001).

## Discussion

A suite of experimental and theoretical techniques was used here to develop structural models for monomeric MB and S-MB, and also dimeric S-MB in lipid-mimic environments. With our molecular dynamics (MD) simulations conducted in 40% HFIP/60% water, the “100 nsec” models for monomeric MB and S-MB (Figs. 4B and 7B) indicated that each peptide is organized as a helical bundle in this membrane-interfacial environment, with S-MB having an additional flexible sequence (residues 1–7) projecting from its core. These monomer simulations were performed using as initial models the residue-specific structures determined from ^13^C-FTIR spectroscopic analyses of the overlapping disulfide-linked MB (PDB: 1SSZ) [48] and/or SP-B(1–25) (PDB: 1DFW) [40] peptides. This general approach has been previously used to investigate MD-simulated interactions between lipids and SP-B(1–25) [101]–[104]. Given the high α-helical levels in the ^12^C-FTIR spectra for either MB or S-MB in both lipids and lipid-mimics (Table 1), the helical bundle structure observed here for “100 nsec” MB and S-MB in 40% HFIP may also be present in the surfactant lipids used in our functional assays. Our subsequent finding that S-MB, but not MB, primarily exists as a dimer in SDS-PAGE (Fig. 8) prompted us to next perform MD simulations to characterize the 3D-structure of dimeric S-MB peptide in SDS and water. Using input structures from ZDOCK and RosettaDock searches, MD simulations produced a “10 nsec” structure (Figs. 11B and 12) confirming the dimer S-MB may bind SDS to form a stable complex in water. Although this “10 nsec” dimer structure is only preliminary because it has not been experimentally verified, such MD simulation models are likely to increase our understanding of S-MB structure and function. In this context, elucidation of the dimer S-MB structure using high-resolution techniques, similar to that conducted earlier from 2D-NMR analysis of monomer MB with SDS micelles (PDB: 2DWF) [49], will be of considerable interest.

It is of interest to compare our “10 nsec” dimer S-MB model with previous experimental structures of related saposin proteins. An approximate two-fold axis lies through the center of the “10 nsec” S-MB dimer, which relates the two leaves each containing the N- and C-terminal α-helices and the N-terminal sequence (1–7) (Figs. 11B and 12A). The space-filling representation in Fig. 12B indicates that the two S-MB monomers join seamlessly to form the “10 nsec” dimer, despite their association being due to non-covalent interactions. SDS molecules predominately bind to an exposed central cavity formed by the hydrophobic residues that line the concave side of the “10 nsec” dimer S-MB model; contrarily, few SDS bind to the opposing convex side that is enriched with polar and positively-charged residues (Figs. 11 and 12). Although the 3D-structure of full-length SP-B has not been experimentally determined, comparisons are possible between our “10 nsec” dimer S-MB and other saposin family members with residue-specific structures. A survey of the experimental saposin structures deposited in the PDB (www.rcsb.org) (see Introduction) indicated that the 2D-NMR spectroscopic structure of saposin C with submicellar SDS (PDB: 1SN6) [20] (Fig. 1) showed several similarities with our “10 nsec” dimer S-MB model (Figs. 11 and 12). For example, each exhibited analogous ‘saposin-like’ folds with their respective leaves in similarly open conformations. The “10 nsec” dimer S-MB model (Figs. 11B and 12) and the saposin C structure (Fig. 1) each have an exposed central cavity formed by the hydrophobic residues that line the concave side, and an opposing convex side with polar and charged residues. Moreover, the external hydrophobic cavities of either the “10 nsec” S-MB model (Figs. 11B and 12) or saposin C [20] were each observed to bind SDS lipid at submicellar detergent concentrations. These correspondences are noteworthy, given that the “10 nsec” dimer S-MB must first self-assemble non-covalently from its monomers, while saposin C has only to fold as a single polypeptide chain.

Our MD simulations indicating that S-MB self-assembles into a stable dimer protein in a SDS-water environment (Figs. 11 and 12) may explain the present findings that S-MB, but not MB, forms dimers in SDS-PAGE (Fig. 8). It is important to note that our SDS-PAGE experiments are not simply conducted in an aqueous buffer, but one that also contains 3.47 mM SDS (i.e., below its CMC of 8.20 mM). Indeed, the basic premise behind MW determinations in SDS-PAGE is that consistent amounts of SDS (∼1.5–2 g detergent/g protein) will typically bind to proteins [91]. Using a similar submicellar SDS concentration in a water environment, our MD simulations indicated that dimer S-MB will actually incorporate a ‘partial micelle’ of SDS to create the final “10 nsec” dimer S-MB ensemble (Figs. 11 and 12). The remarkable formation of a stable, globular lipoprotein from constituent S-MB and SDS is attributed to the N-terminal residues (1–7) in S-MB (Fig. 2), as analogous dimers are not observed in SDS-PAGE of MB (Fig. 8). Our MD simulations suggest that the N-terminal insertion sequence is critical for maintaining the dimer S-MB structure, because this hydrophobic sequence and the bundle of N- and C-terminal helices together make up the interior surface of the exposed cavity which binds SDS (see below). Conceivably, an analogous dimer S-MB model may also account for the strong binding of S-MB to itself seen in SPR studies (Fig. 10; Table 2). Comparable to the SDS-PAGE experiments, SPR binding assays are performed in an aqueous buffer that includes 4.08 mM polysorbate 20 (P20) (i.e., below its CMC of 4.89 mM). P20 is an amphipathic detergent, which like SDS has been used as a solubilizing agent to extract membrane proteins [105]. In a manner similar to that described for SDS (Figs. 11 and 12), P20 molecules may promote the assembly of the dimer S-MB complex by forming a ‘partial micelle’ in the central cavity formed by the two S-MB molecules. It is also of interest that strong interactions were noted for S-MB with itself, but not for the self-association of MB or the cross-association of MB with S-MB (Fig. 10; Table 2). This suggests that both N-terminal insertion sequences must be present in the cavity of the dimer peptide, followed by incorporation with the P20 ‘partial micelle’, before strong intermolecular associations will form between homodimers and/or heterodimers (see below).

Although the “10 nsec” MD simulation of dimer S-MB provides a reasonable framework to account for our SDS-PAGE and SPR results (Figs. 8 and 10), it is worthwhile to consider other structural models by which the N-terminal insertion sequence may promote the self-association of S-MB. One possible alternative is that oligomeric S-MB may be due to the N-terminal sequence (residues 1-7) forming β-sheet. The N-terminal SP-B(1–9) (Fig. 2) was earlier proposed to act as a ‘biochemical Velcro®,’ facilitating *in vivo* either the aggregation of SP-B or the interactions of SP-B with SP-C [50]. In support of this model were earlier ^13^C-FTIR spectroscopic studies of SP-B(1–25) in POPG liposomes, which indicated that residues 1–5 participated in interpeptide β-sheet [40]. Nevertheless, most theoretical and experimental evidence argues against the N-terminal sequence (1–7) by itself promoting S-MB oligomers. SP-B(1-7) does not form a standard Pauling-Corey β-sheet on Ramachandran analysis, but instead belongs to a broader class of extended conformations that includes the β-sheet [97]. If anything, the repeated proline motif should act as a β-sheet ‘breaker’ [97], [106], due to each proline residue lacking the amide hydrogen and structural constraints imposed by its pyrrolidine ring. Consistent with this are PASTA calculations indicating high energies (i.e., low aggregation) for the best pairings of SP-B(1–7) (i.e., respective PASTA relative energies of 1.94 for parallel segments 1–5, and 2.88 for antiparallel segments 4–7) [62]. Also in support of the N-terminal residues (1–7) not participating in β-sheet is the earlier FTIR spectral result indicating only minor β-sheet (i.e., 26.3% or ∼2.4 residues) for the overlapping SP-B(1–9) peptide in methanol, a mimic of the lipid-water interface [30]. Another mechanism by which the N-terminal sequence (1–7) might promote S-MB oligomer formation is through relatively non-specific aggregation of its hydrophobic sidechains, particularly in water. Interestingly, earlier ESR studies of the overlapping SP-B(1–25), spin-labeled at the amino-terminal Phe-1, demonstrated that this peptide was primarily aggregated in PBS, exhibiting spectra that were not only exchange-broadened and motionally-restricted, but also insensitive to the paramagnetic broadening agent chromium oxalate [30]. These ESR results indicated that spin-labeled SP-B(1–25) formed high MW oligomers, possibly involving the formation of a peptide-like micelle with the spin-label reporter group buried in the interior of the peptide aggregate. However, it should also be noted that addition of SDS micelles rapidly dissociated these SP-B(1–25) aggregates, producing a dilute ESR spectrum indicating insertion of the spin-labeled N-terminal SP-B(1–25) peptide into the SDS micelle. The above non-specific aggregation mechanism is unlikely to account for the dimer formation of S-MB in SDS-PAGE for several reasons. First, the SDS-PAGE in Fig. 8 is not performed in an aqueous buffer, but one that has high SDS levels that will disaggregate any non-specific oligomers of S-MB, analogous to that observed for the overlapping spin-labeled SP-B(1–25) [30]. Second, S-MB dimers based on non-specific hydrophobic interactions between the N-terminal 1–7 residues are improbable, because such configurations were not among the ten lowest energy conformers of the 1000 decoy candidates tested with RosettaDock (see above). Last, our “10 nsec” MD simulation for dimer S-MB in SDS-water (Figs. 11 and 12) indicated no conformational drift to this alternative model, which was subsequently confirmed by extending this MD simulation run to 50 nsec (data not shown).

Synthetic lung surfactant preparations containing synthetic lipids plus S-MB or MB had high surface activity in captive bubble surfactometer experiments, and also improved pulmonary function and mechanics in ventilated lung-lavaged rats with ARDS to an even greater extent than porcine SP-B surfactant. These findings with synthetic lipid/peptide surfactants are consistent with our recent reports detailing not only enhanced surfactant activity with MB and typical surfactant lipids in *in vitro* and *in vivo* experiments [48], but also high surface activity and inhibition resistance of a synthetic surfactant preparation containing MB peptide combined at 1.5% by weight with a phospholipase-resistant phosphonolipid compound (DEPN-8) [16]. These earlier results, coupled with the surface and physiological activity data found here for S-MB and MB surfactants, strongly support the use of SP-B-related peptides in synthetic lipid/peptide exogenous surfactants for treating lung surfactant deficiency (NRDS) or injury-induced dysfunction (ALI/ARDS). As the S-MB peptide investigated here had even greater pulmonary activity than MB, S-MB may be preferred over MB in totally-synthetic surfactant preparations also containing synthetic lipids.

The active components of endogenous surfactant and all current exogenous surfactants are lipids and peptides, and this also is the case for the synthetic surfactants of this study. Endogenous surfactant contains a complex mixture of lipids, with a predominant phospholipid content of 85–90% by weight and cholesterol content of 4–5% by weight ([8] for review). The composition of synthetic lipids in synthetic surfactants here was a mixture of five components (DPPC, POPC, POPG, POPE and cholesterol) that together accounted for the major lipid-based molecular interactions in native surfactant. In terms of chain-chain interactions, the four phospholipids in this lipid mixture incorporated intermolecular biophysical interactions between either identical C16∶0 acyl chains or between C16∶0 and C16∶1 acyl chains. Endogenous surfactant phospholipids contain a substantial content of both these fatty acyl moieties [8], [107], [108]. In addition, the phospholipids in surfactant lipids allowed for molecular interactions involving both zwitterionic (PC) and anionic (PG) headgroups that are prominent in native surfactant. This includes not only headgroup/headgroup interactions among lipid molecules themselves, but also lipid headgroup interactions with charged or polar amino acids in peptides. DOPE and cholesterol were present in surfactant lipid in smaller amounts than other lipids, but contributed additional molecular features. Because of the small size of the PE headgroup relative to PC, the DOPE molecule has a more wedge-shaped cross-section that affects molecular packing in lipid bilayers and films. Cholesterol also has the ability to influence local fluidity/rigidity and packing in lipid membranes and films [8], [109], [110] and may additionally increase lipid adsorption [8].

The biophysical behavior of lipids in endogenous surfactant is significantly increased by molecular interactions with three active apoproteins (SP-A, -B and -C). All these apoproteins interact strongly with lipids at the molecular level [8], [111], [112], but peptides related to SP-B are of special interest because it is known to be particularly active in improving the adsorption and film behavior of lung surfactant lipids [8], [13], [17]. In the present studies, MB and S-MB were designed to maintain key structural features of the full-length human SP-B [48]. The N- and C-terminal domains of native SP-B actively bind lipids [30], [34], [39], [40], [44], and MB and S-MB each incorporates residues 8–25 and 63–78 of human SP-B that participate in these amphipathic helices (Figs. 4,6,7). These N- and C-terminal regions are joined in either MB or S-MB by means of a novel loop domain [16], [48], which simulates that occurring in full-length SP-B [55]. Peptide folding during synthesis is facilitated by specific solvents to produce the necessary helix-hairpin structure stabilized by oxidation of cysteine residues, allowing MB and S-MB to form intramolecular disulfide connectivities analogous to those between Cys-8 and Cys-78 and Cys-11 and Cys-71 in human SP-B (residue numbers refer to the full-length sequence of SP-B) [48]. As reviewed previously [48], earlier theoretical and physical studies on peptides based on the N- and C-terminal domains of SP-B suggest that the cross-linked, amphipathic helical domains of MB partition into the polar headgroup region of lipids. Consistent with this are SPR results indicating that MB will bind to either DPPC or the synthetic lipid DEPN-8 with high affinity [16]. Given that MB and S-MB in lipids and lipid-mimics show similarly high α-helix on FTIR analysis (Fig. 3 and Table 1), and also demonstrate comparable helical bundles for their shared residues in the lipid-mimic 40% HFIP (Figs. 4,6,7), it is reasonable to propose that the adjacent, positively-charged helices in S-MB will also be surface-seeking in surfactant lipids.

Besides promoting the formation of a dimer S-MB that assumes a ‘saposin-like’ fold (Figs. 11 and 12), the N-terminal insertion sequence with its hydrophobic residues (Fig. 2) may also anchor the S-MB helices onto surfactant lipids, as has been noted in earlier physical [30] and MD simulation [103] studies of the overlapping SP-B(1–25) peptide. Such a dual structural role for the N-terminal insertion sequence may explain the dramatic enhancement of *in vivo* surfactant activities observed here for S-MB (Fig. 14). The present results support the basic concept that the N-terminal SP-B(1–9) may act as a ‘biochemical Velcro®,’ promoting the *in vivo* aggregation of SP-B or the interactions of SP-B with SP-C [50]. Based on our PASTA predictions and docking searches (see above), however, the self-adhesive region was identified as the overlapping Tyr-7 to Arg-12 sequence, which likely forms an antiparallel β-sheet in aqueous environments. The extended conformation for dimer S-MB, obtained from the lowest energy RosettaDock searches (e.g., the “0 nsec” model in Fig. 11A), may reflect the dimer peptide in polar environments, such as the aqueous buffer or at the lipid-water interface.

It is of particular interest that the “10 nsec” model for the dimer peptide in SDS (Figs. 11B and 12) may represent membrane-bound dimer S-MB, where the dimer adopts a relatively open ‘saposin-like’ conformation when interacting with membrane lipids. Before making this assignment, however, it is important to first assess whether SDS is a useful surrogate for the typical phospholipids in membrane lipids. To test this hypothesis, control FTIR experiments were performed to compare the secondary conformations of MB and S-MB in SDS. Fig. 3 and Table 1 indicate similar % secondary conformations for these peptides in either SDS or phospholipids, consistent with SDS being a reasonable substitute. It should also be noted that previous ESR experiments using the spin-labeled N-terminus of SP-B(1–25) indicated comparable incorporation of the N-terminal Phe of SP-B(1–25) into either SDS micelles or phospholipid bilayers [30]. Moreover, the 3D-structure of MB in POPG liposomes determined from residue-specific ^13^C-FTIR spectroscopy [48] (PDB: 1SSZ) was very similar to that obtained from 2D-NMR analysis of MB in SDS micelles [49] (PDB: 2DWF). These latter findings are consistent with prior 2D-NMR analyses suggesting that membrane proteins may adopt native conformations when incorporated into SDS [20], [113]. Collectively, these findings from control experiments indicate that SDS may be a reasonable first approximation of membrane phospholipids. Our “10 nsec” model (Figs. 11B and 12) shows that dimer S-MB integrates a partial SDS micelle into its structure, which then separates the antiparallel β-sheet pairing (i.e., Tyr-7 to Arg-12). Each monomer in the “10 nsec” dimer S-MB model optimizes its interactions with SDS, yet the overall organization of the dimer S-MB and its local two-fold axis are still retained. Thus, the “10 nsec” dimer S-MB model (Figs. 11B and 12) may be the surfactant-active configuration, which inserts into the membrane so that its N-terminal sequences (1–7) are immediately subjacent to the polar lipid headgroup, and its amphipathic N- and C-terminal helices are bound at the lipid-water interface with their hydrophobic faces oriented inward [30], [44], [97], [103]. In this context, it should be noted that recent coaxial confocal-AFM imaging and FRET assays [114] suggest that saposin C may perturb membrane bilayers by adopting an open conformation, thereby exposing its inner hydrophobic surfaces for interactions with lipid acyl chains. Because both saposin C (Fig. 1) [20] and the “10 nsec” dimer S-MB model (Figs. 11B and 12) share an open, ‘saposin-like’ conformation when bound to submicellar SDS, it is tempting to speculate that dimer S-MB may exert its surfactant activities by analogously binding to lipid bilayers and monolayers. The above results also indicate not only that the extended N-terminal insertion sequence (residues 1–12) anchors the dimer S-MB in lipids and promotes the self-association of S-MB, but also that these two structural properties may be antagonistic to some extent. Conceivably, the expression of optimal surfactant activities produced by either S-MB or native SP-B proteins may depend on the N-terminal sequence (1–12) achieving a finely-tuned balance between protein self-association and insertion into lipids.

Functional and structural studies in our laboratory are continuing to investigate the surfactant properties of additional SP-B peptide mimics, including covalently-linked dimer forms (e.g., Ref. [36]), SP-B constructs containing extended regions of the full-human sequence, and SP-B variants with modified sequences. Here, we report that the high *in vivo* surfactant activity for S-MB may be partially due to the ability of this peptide to form non-covalently associated dimers. These findings are broadly supportive of previous experiments indicating that the dimeric full-length SP-B, associated through either covalent or non-covalent linkages, shows elevated *in vitro* and *in vivo* surfactant activities over those of the monomeric protein [92], [115]. Although the present experiments demonstrated higher *in vivo* surfactant activity for S-MB than for native SP-B (Fig. 14), our *in vitro* results also indicated that S-MB does not completely reproduce all of the surface-tension lowering properties of full-length SP-B in captive bubble surfactometry (Fig. 13). The discrepancies between our *in vivo* and *in vitro* findings suggest that further modifications of S-MB may be required to obtain a fully-optimized SP-B mimic.

In summary, FTIR spectroscopy of S-MB and MB in lipids and lipid-mimics showed that these peptides exhibit similar conformations, with primary α-helix and secondary β-sheet and loop-turns. With each peptide treated as a monomer in a lipid-mimic environment, subsequent MD simulations indicated that S-MB and MB not only share the same bundle of adjacent N- and C-terminal α-helical domains [48], [49], but also that the N-terminal insertion sequence (residues 1–7) of S-MB assumes an extended conformation projecting from its helical core. SDS-PAGE electrophoresis demonstrated that S-MB was dimeric in submicellar SDS concentrations, while MB was monomeric. SPR, predictive aggregation algorithms, and MD and docking simulations further suggested a preliminary model for dimeric S-MB with SDS, in which monomers non-covalently associate to form a dimer S-MB peptide with a ‘saposin-like’ fold in both aqueous and lipid environments. The external N-terminal insertion domain (residues 1–12) may act as a ‘biochemical Velcro®’ [50] to fasten S-MB molecules together into a dimer peptide that adopts the open saposin-fold when bound to lipids. These membrane-associated dimer S-MB peptides may possibly self-associate to form protein-rich networks in lipids, analogous to those observed for native SP-B and other SP-B peptides containing the insertion sequence [32], [116]. Besides promoting peptide self-association, these and prior investigations with SP-B analogs indicate that the hydrophobic N-terminal insertion sequence may assist in anchoring S-MB into lipid bilayers and monolayers. *In vitro* and *in vivo* experiments also indicated that S-MB and MB each exhibits a range of surfactant activities, with S-MB showing greater oxygenation and dynamic compliance in animal models than MB. Consequently, our functional studies are supportive of earlier results with SP-B peptides and peptide analogs, which demonstrated that this N-terminal insertion sequence plays critical roles in the expression of *in vitro* surfactant activities [38], [52], [53].
